# Genome-wide identification and analysis of the *WUSCHEL*-related homeobox (*WOX*) gene family in allotetraploid *Brassica napus* reveals changes in *WOX* genes during polyploidization

**DOI:** 10.1186/s12864-019-5684-3

**Published:** 2019-04-25

**Authors:** Mengdi Li, Ruihua Wang, Zhengyi Liu, Xiaoming Wu, Jianbo Wang

**Affiliations:** 1 State Key Laboratory of Hybrid Rice, College of Life Sciences, Wuhan University, Wuhan, 430072 China; 2 Key Laboratory of Biology and Genetic Improvement of Oil Crops, Ministry of Agriculture, Oil Crops Research Institute of CAAS, Wuhan, 430062 China

**Keywords:** *WUSCHEL*-related homeobox gene, *WOX* gene family, Homeobox domain, Allotetraploid, *Brassica napus*, Polyploidization, Expression pattern

## Abstract

**Background:**

*WUSCHEL*-related homeobox (*WOX*) genes encoding plant-specific homeobox (HB) transcription factors play important roles in the growth and development of plants. To date, *WOX* genes has been identified and analyzed in many polyploids (such as cotton and tobacco), but the evolutionary analysis of them during polyploidization is rare. With the completion of genome sequencing, allotetraploid *Brassica napus* and its diploid progenitors (*B. rapa* and *B. oleracea*) are a good system for studying this question.

**Results:**

In this study, 52, 25 and 29 *WOX* genes were identified in allotetraploid *B. napus* (2n = 4x = 38, A_n_C_n_), the A_n_ genome donor *B. rapa* (2n = 2x = 20, A_r_) and the C_n_ genome donor *B. oleracea* (2n = 2x = 18, C_o_), respectively. All identified *WOX* genes in *B. napus* and its diploid progenitors were divided into three clades, and these genes were selected to perform gene structure and chromosome location analysis. The results showed that at least 70 and 67% of *WOX* genes maintained the same gene structure and relative position on chromosomes, respectively, indicating that *WOX* genes in *B. napus* were highly conserved at the DNA level during polyploidization. In addition, the analysis of duplicated genes and transposable elements (TEs) near *WOX* genes showed that whole-genome triplication (WGT) events, segmental duplication and abundant TEs played important roles in the expansion of the *WOX* gene family in *B. napus*. Moreover, the analysis of the expression profiles of *WOX* gene pairs with evolutionary relationships suggested that the *WOX* gene family may have changed at the transcriptional regulation level during polyploidization.

**Conclusions:**

The results of this study increased our understanding of the *WOX* genes in *B. napus* and its diploid progenitors, providing a rich resource for further study of *WOX* genes in these species. In addition, the changes in *WOX* genes during the process of polyploidization were discussed from the aspects of gene number, gene structure, gene relative location and gene expression, which provides a reference for future polyploidization analysis.

**Electronic supplementary material:**

The online version of this article (10.1186/s12864-019-5684-3) contains supplementary material, which is available to authorized users.

## Background

The superfamily of HB transcription factors is a large family with many members in eukaryotes [1]. A common feature of proteins in this superfamily is that they contain a homeobox domain that consists of 60–66 amino acids folded into a helix-turn-helix structure, which can be recognized by specific DNA to regulate target gene expression at a precise moment [1, 2]. In this HB superfamily, *WOX* genes encode plant-specific HB transcription factors [3]. Previous studies have found that *WOX* genes are present in the genomes of many plants, ranging from lower plants, such as green algae, to higher plants, such as angiosperms [2]. The number of *WOX* genes varies from plant to plant, and the *WOX* gene family has gradually expanded with the continuous evolution of plants; for example, there is just one *WOX* gene in unicellular green algae, three in moss, six in Selaginella, and 15 in Arabidopsis [4]. The *WOX* gene family can be divided into three clades, the ancient clade, intermediate clade and WUS clade, by phylogenetic analysis of *WOX* genes in different plants [5, 6]. The *WOX* genes of lower plants belong only to the ancient clade, while those of higher plants belong to all three clades [7].

Studies have shown that *WOX* genes play crucial roles in the growth and development of plants, such as stem cell regulation [8], embryo patterning [9], and flower development [10]. The functions and characteristics of *WOX* genes have been well studied in the typical model plant *Arabidopsis thaliana*, in which 15 *WOX* genes have been identified [6, 7]. *WUS*, which is expressed in the ovule, anther, and shoot apical meristem, has been shown to act a pivotal part in central meristem maintenance [11]. Overexpression of *WOX1* causes abnormal meristem in *A. thaliana* [12]. WOX2 and WOX8 have a critical function in early embryo patterning, and they are expressed in the zygote and then confined to the apical and basal cell, respectively [13, 14]. WOX3 was found to participate in the formation of lateral and marginal regions of vegetative and floral organs [15]. WOX4, in coordination with PXY, works in auxin-dependent cambium stimulation to regulate lateral plant growth [16]. WOX5 was demonstrated to act as a vital regulator in the root apical meristem, which is necessary for forming the correct root pattern [17]. WOX6/PFS2 was shown to regulate ovule development and affect ovule patterning [18]. *WOX7*, which is expressed in lateral roots, was confirmed to inhibit the development of lateral roots in a sugar-dependent manner [19]. STIMPY/WOX9 integrates developmental signals and cell cycle regulation to maintain cell division and prevent inappropriate differentiation in roots [20]. WOX11 was determined to be involved in a process in which some vascular cambium initially is converted to new lateral root founder cells [21]. WOX11 and its homolog WOX12 were found to participate in de novo root organogenesis [22]. WOX13 promotes replum formation and regulates fruit patterning during fruit development [23]. WOX14 promoted vascular cell lignification by increasing the accumulation of bioactive gibberellin (GA) in the inflorescence stems of Arabidopsis [24].

*Brassica napus*, an allotetraploid of the *Brassica* genus, is a considerable oil crop planted worldwide. According to previous studies, the WGT event occurred in ancestors of the *Brassica* genus ~ 15.9 million years ago (MYA) [25]. Then, diploid *B. rapa* (2n = 20, A_r_) and *B. oleracea* (2n = 18, C_o_) were successively formed ~ 4.6 MYA [25]. Finally, *B. napus* (2n = 4x = 38, A_n_C_n_) was formed by natural hybridization and polyploidization of *B. rapa* and *B. oleracea* ~ 7500 years ago [25]. The genomes of *B. napus* (cv. Darmor-*bzh*), *B. rapa* (cv. Chiifu-401-42) and *B. oleracea* (var. *capitata*-02-12) have already been sequenced and assembled [25–27]. Thus, the natural allotetraploid *B. napus* and its diploid progenitors (*B. rapa* and *B. oleracea*) were always used to study the scientific problems associated with polyploidization. So far, *WOX* genes has been identified and analyzed in many polyploids (such as cotton [5] and tobacco [6]), but the evolutionary analysis of them during polyploidization is rare. Therefore, we described *WOX* genes systematically in *B. napus* and its diploid progenitors and hoped to find insights regarding *WOX* genes during polyploidization. This study included several parts, including the identification of the *WOX* gene family and gene structure, conserved domain analysis, phylogenetic tree analysis, chromosomal localization analysis, synteny and duplicated gene analysis, and expression pattern analysis.

## Results

### Identification and characterization of *WOX* genes

To identify the putative *WOX* genes in *B. napus* and its diploid progenitors, 15 WOX protein sequences of Arabidopsis were acquired and used as query sequences to search against the BRAD database [28] using the BLASTp program [29]. As a result, 28, 24 and 62 genes were selected as original candidate genes in *B. rapa*, *B. oleracea* and *B. napus*, respectively. Then, the syntenic genes were searched in the BRAD database by inputting the gene IDs of the *WOX* genes in Arabidopsis, which is a supplement for the first method. As a result, an additional five genes were also identified as *WOX* genes in *B. oleracea*. Then, three public protein databases (Pfam, SMART and CDD database) were used to search the HB domain in protein sequences encoded by candidate *WOX* genes, and proteins that did not contain the complete conserved HB domain were removed. Finally, 25, 29 and 52 genes were identified as *WOX* genes in *B. rapa*, *B. oleracea* and *B. napus*, respectively. It was clear that the total *WOX* genes in two diploid progenitors, *B. rapa* and *B. oleracea*, was higher than that in the allotetraploid *B. napus*, which indicated that a gene loss event might have occurred in the *WOX* gene family of *B. napus* during polyploidization.

These identified *WOX* genes in *B. napus* and its diploid progenitors were named, i.e., from *BrWUSa* to *BrWOX14b* in *B. rapa*, *BoWUSa* to *BoWOX14c* in *B. oleracea* and *BnAWUSa* to *BnAWOX14e* in *B. napus*, according to the homologous relationship with corresponding *WOX* genes in Arabidopsis (Additional file 1: Table S1). The last lowercase letter in the name represents the degree of homology to the corresponding gene in Arabidopsis, with ‘*a*’ representing the highest homology, followed by ‘*b*’, and so on. In *B. napus*, the capital letters *A* and *C* following ‘*Bn*’ represent the A_n_ and C_n_ subgenomes, respectively. The length of the WOX protein sequences ranged from 133 (BnAWOX14e) to 397 (BnCWOX9a) amino acids in *B. napus*. In addition, the physical and chemical characteristics of a total of 106 WOX proteins were analyzed and provided, including the molecular weights (MW), theoretical PI values, instability index (II), grand average of hydropathicity (GRAVY) and aliphatic index (Additional file 1: Table S1). The average values of these physical and chemical characteristics were approximately equal to each other in *B. napus* and its diploid progenitors upon calculation.

### Phylogenetic analysis and gene structure analysis

**Fig. 1:**
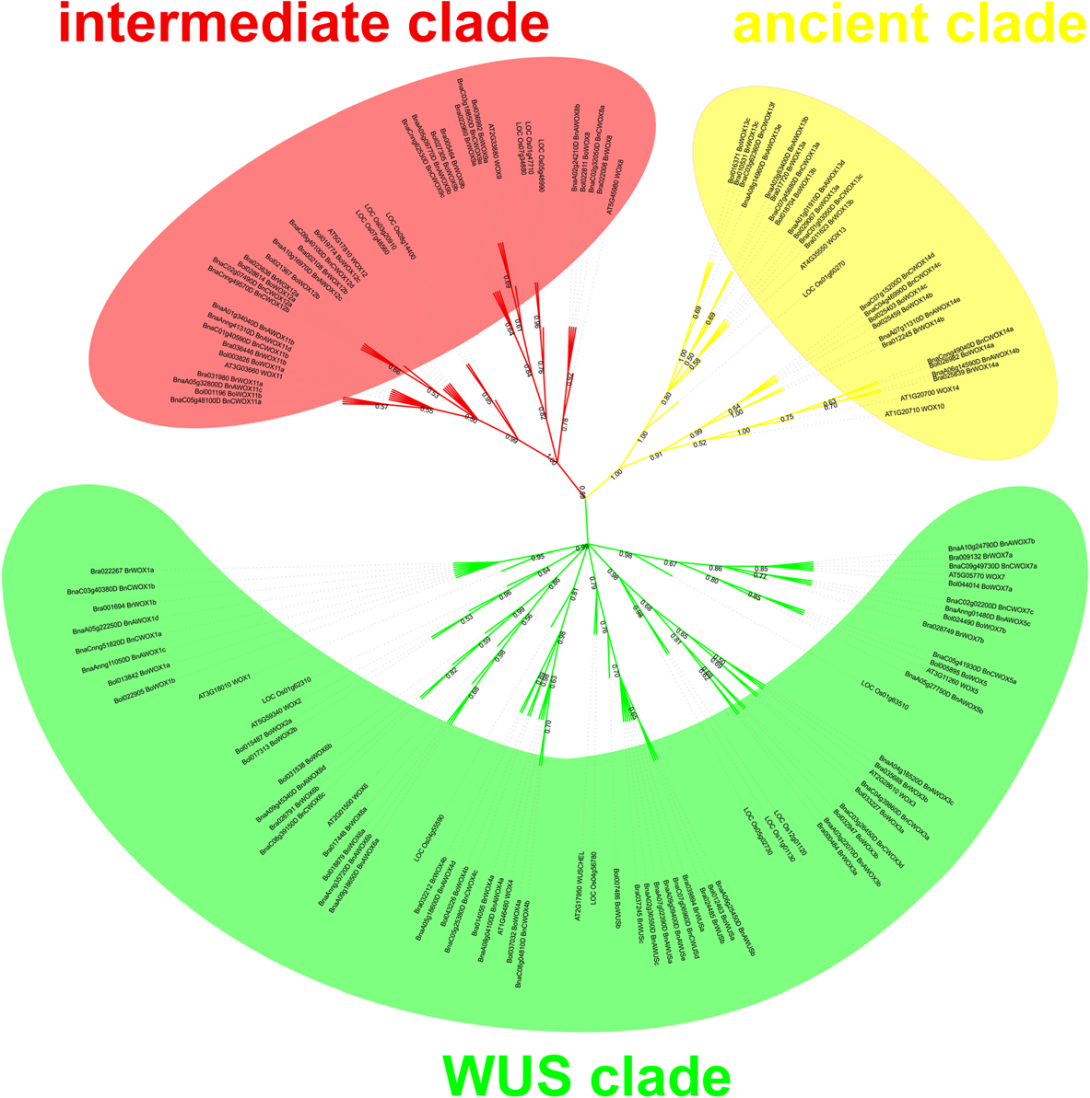
Phylogenetic tree of WOX proteins in *B. rapa*, *B. oleracea*, *B. napus*, Arabidopsis and rice. This tree could be divided into the ancient clade (yellow), intermediate clade (red) and WUS clade (green). This phylogenetic unrooted tree was constructed using MEGA7.0 with 1000 bootstrap replicates and only those values greater than 50% are displayed

WOX proteins from typical monocots (rice) and dicots (Arabidopsis) were used as reference proteins to construct the WOX phylogenetic tree, where WOX proteins in rice were identified by the same methods mentioned above. Therefore, the unrooted phylogenetic tree was constructed based on a total of 135 WOX protein sequences, including 15 in Arabidopsis, 25 in *B. rapa*, 29 in *B. oleracea*, 52 in *B. napus* and 14 in rice members (Fig. 1). Evidently, the phylogenetic tree showed that WOX proteins were classified into three clades, which were the ancient clade, intermediate clade and WUS clade. According to statistical analysis, the number of WOX proteins in the WUS clade (70) was greater than the sum of proteins in the ancient clade (26) and the intermediate clade (39). Hence, the WUS clade was the largest clade of the WOX proteins in these five species. Notably, WOX proteins in *B. napus* and its diploid progenitors were related to their corresponding homologs in Arabidopsis or rice in each clade, which suggested that the evolutionary relationship of WOX transcription factors is very close in these species.

**Fig. 2:**
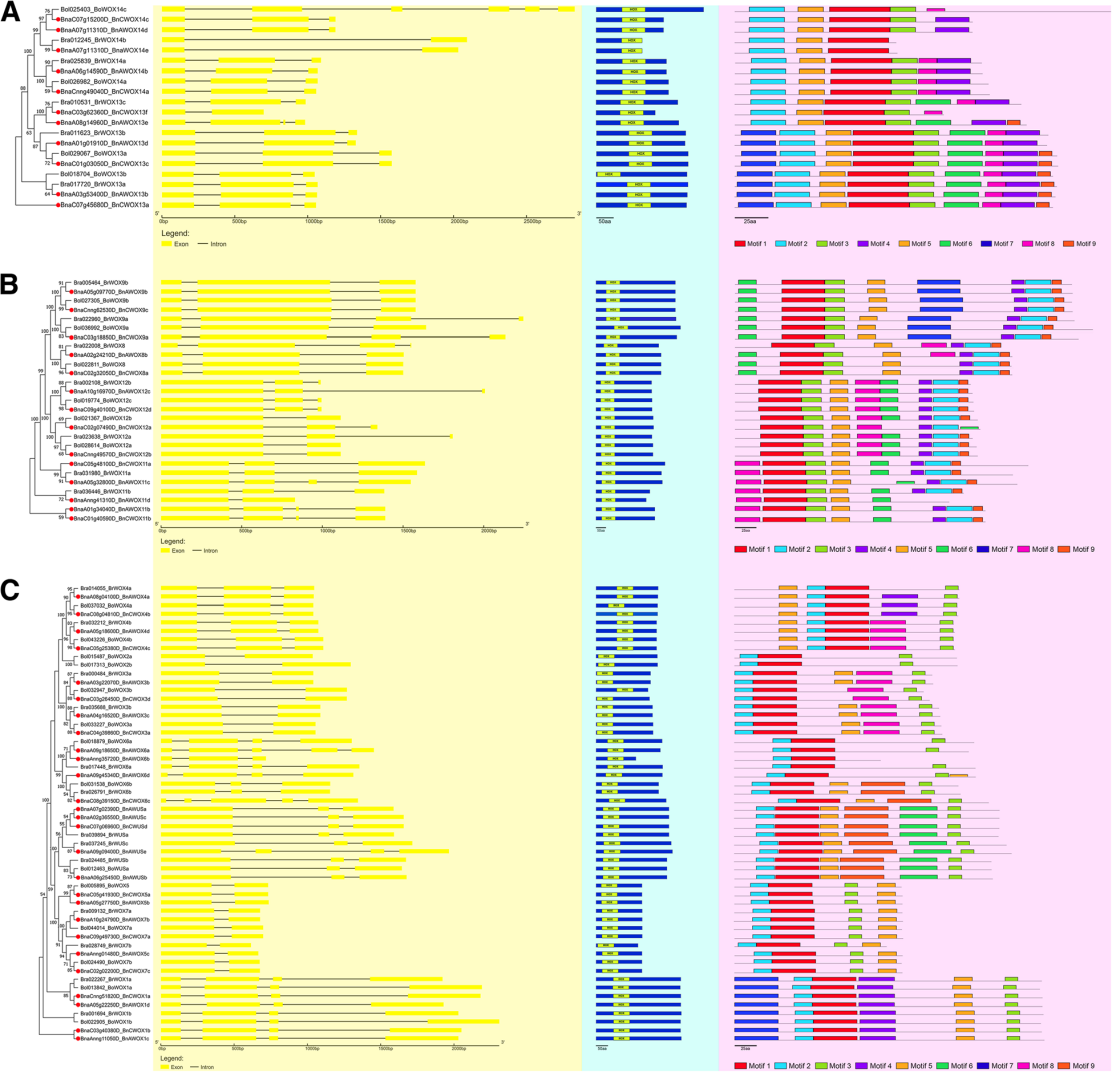
Characterizations of the identified *WOXs* in *B. napus* and its diploid progenitors. The characterizations include intron/exon structure (yellow background), domain location (blue background) and conserved motif location (purple background). **a** the characterizations of the *WOX* genes in the ancient clade. **b** the characterizations of the *WOX* genes in the intermediate clade. **c** the characterizations of the *WOX* genes in WUS clade. The *WOX* genes in *B. napus* were marked by the red circle

For exploring more characteristics about WOX proteins in each clade, WOX protein sequences within the three clades were selected separately to build three phylogenetic trees (Fig. 2). The protein sequences in each clade were similar to each other, whether they were proteins among allotetraploid *B. napus* or its diploid progenitors. The ancient clade consisted of WOX13 and WOX14, while the intermediate clade consisted of WOX8, WOX9, WOX11, and WOX12, and the WUS clade consisted of WUS and WOX1–7. Interestingly, the homolog of WOX10 could not be found either in *B. napus* or its diploid progenitors*.* In addition, exon/intron structures were analyzed to show the structural diversity of *WOX* genes in different clades and to explore whether the gene structure changed during the polyploidization process (Fig. 2). The results showed that most of the genes had three exons in both the ancient clade and intermediate clade, while 21 genes had two exons, and 19 genes had three exons in WUS clade. By comparison, we found that the gene structure of *WOX* genes from the WUS clade was significantly more conserved than that of the other two clades during allotetraploid *B. napus* formation. Six out of eight kinds of *WOX* genes in the WUS clade had the same gene structure, namely, *WOX1*, *WOX2*, *WOX3*, *WOX4*, *WOX5* and *WOX7*, whether they were from allotetraploid *B. napus* or its diploid progenitors. In addition, if two genes that came from the allotetraploid and one of its two diploid progenitors branched at the same final level in the phylogenetic tree, they may have a direct evolutionary relationship. Statistical analysis showed that a total of 33 pairs of *WOX* genes were found that may have direct evolutionary relationships in these three phylogenetic trees (Table 1). Five out of seven pairs of *WOX* genes (approximately 71%) in the ancient clade, six out of eleven pairs (approximately 55%) in the intermediate clade and 12 out of 15 pairs (80%) in WUS clade had the same number of exons (Table 1). Thus, 23 out of the 33 gene pairs (approximately 70%) maintained the same gene structure during the formation of *B. napus*. Therefore, *WOX* genes were conserved at the DNA level during polyploidization. Furthermore, the location of the HB domain was visualized to facilitate the analysis of the changes in the domain’s position between different clades or different species (Fig. 2). The HB domain of many WOX proteins was located in the N-terminus of the protein in both the intermediate and WUS clades but was located in the middle part of the protein in the ancient clade. We could also see that the length and position of the HB domain were generally conserved. In addition, the MEME website was used to predict conserved motifs in WOX proteins (Fig. 2), and the results showed that at most nine motifs were found in WOX proteins, and only motif 1 was found in every WOX. In general, the *WOX* gene family in *B. napus* and its diploid progenitors was very conserved at the DNA and protein level, which might be related to the important function of the *WOX* genes in these species.

**Table 1:** Information about *WOX* gene pairs with potential direct evolutionary relationships

Clade	*WOX* genes in diploid progenitors		*WOX* genes in allotetraploid *B. napus*	
	Gene name	No. of exons	Gene name	No. of exons
Ancient clade	*BoWOX14c*	6	*BnCWOX14c*	3
	*BrWOX14b*	2	*BnAWOX14e*	2
	*BrWOX14a*	3	*BnAWOX14b*	3
	*BoWOX14a*	3	*BnCWOX14a*	3
	*BrWOX13c*	3	*BnCWOX13f*	2
	*BoWOX13a*	3	*BnCWOX13c*	3
	*BrWOX13a*	3	*BnAWOX13b*	3
Intermediate clade	*BrWOX9b*	3	*BnAWOX9b*	3
	*BoWOX9b*	3	*BnCWOX9c*	3
	*BoWOX9a*	3	*BnCWOX9a*	4
	*BrWOX8*	4	*BnAWOX8b*	3
	*BoWOX8*	3	*BnCWOX8a*	3
	*BrWOX12b*	3	*BnAWOX12c*	3
	*BoWOX12c*	3	*BnCWOX12d*	3
	*BoWOX12b*	2	*BnCWOX12a*	3
	*BoWOX12a*	2	*BnCWOX12b*	2
	*BrWOX11a*	3	*BnAWOX11c*	4
	*BrWOX11b*	3	*BnAWOX11d*	2
WUS clade	*BrWOX4a*	3	*BnAWOX4a*	3
	*BoWOX4a*	3	*BnCWOX4b*	3
	*BrWOX4b*	3	*BnAWOX4d*	3
	*BoWOX4b*	3	*BnCWOX4c*	3
	*BrWOX3a*	2	*BnAWOX3b*	2
	*BoWOX3b*	2	*BnCWOX3d*	2
	*BrWOX3b*	2	*BnAWOX3c*	2
	*BoWOX3a*	2	*BnCWOX3a*	2
	*BoWOX6a*	4	*BnAWOX6a*	5
	*BrWOX6b*	3	*BnCWOX6c*	5
	*BrWUSc*	3	*BnAWUSe*	4
	*BoWUSa*	3	*BnAWUSb*	3
	*BoWOX5*	2	*BnCWOX5a*	2
	*BrWOX7a*	2	*BnAWOX7b*	2
	*BoWOX7b*	2	*BnCWOX7c*	2

### Conserved amino acid sequences within the homeobox domain

**Fig. 3:**
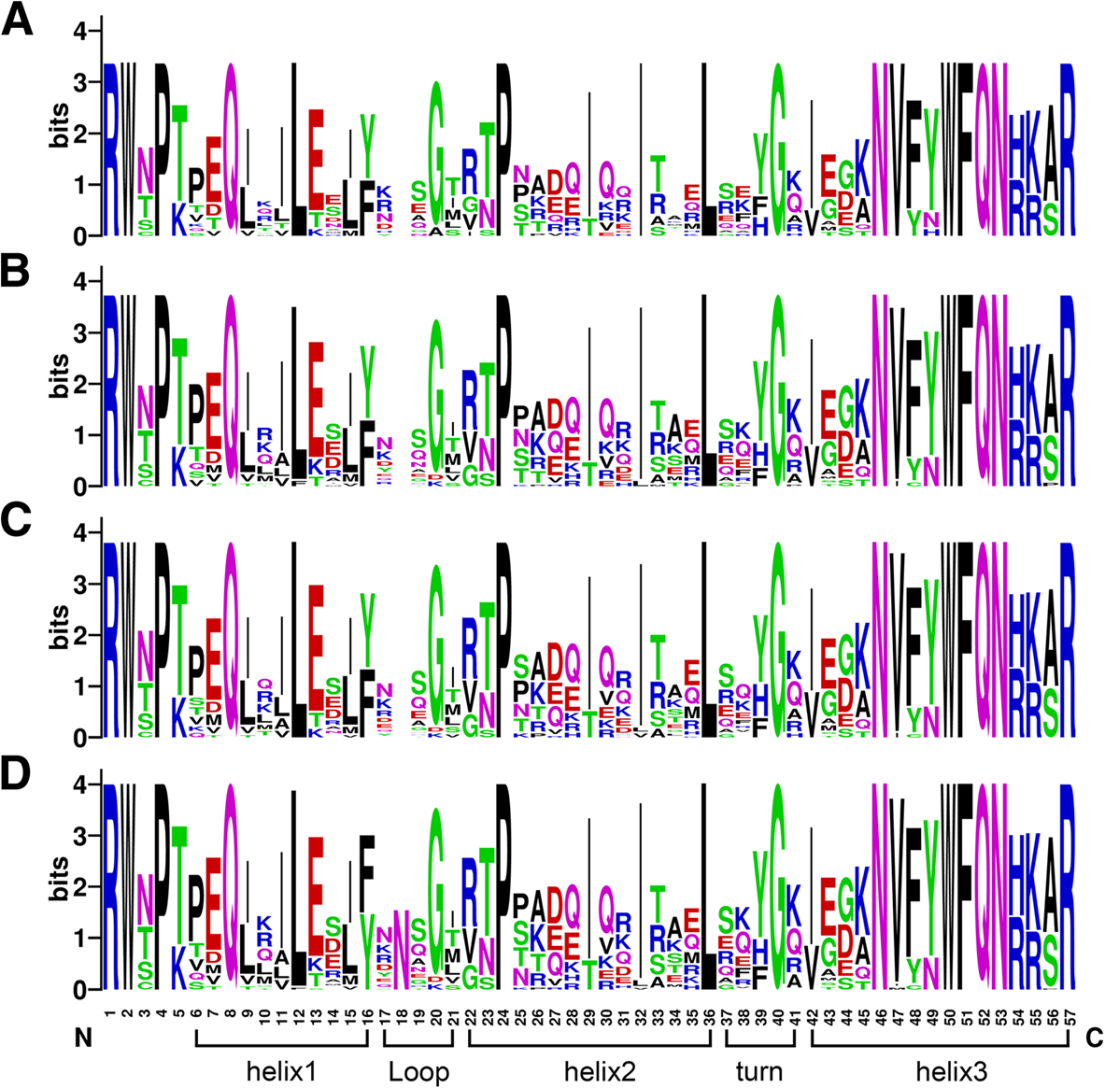
Sequence logos about the homeobox domain. **a** The logo in Arabidopsis. **b** The logo in *B. rapa*. **c** The logo in *B. oleracea*. **d** The logo in *B. napus*. This domain contained a total of 57 amino acids, including one loop, one turn and three helix structures

The *WOX* gene family is a plant-specific gene family, of which the typical characteristic is that every WOX protein encoded has a completely conserved HB domain [1, 2]. To study the sequence of the conserved HB domains and the degree of their conservation in different Brassicaceae species, multiple sequence alignment was used to generate the protein sequence logos in *B. rapa, B. oleracea, B. napus* and Arabidopsis (Fig. 3). The sequence logos showed that the amino acids and their distribution in the HB domain were remarkably similar in these four plants. The HB domain contained one loop, one turn and three helix structures and consisted of 57 amino acids, which was consistent with previous research results [5]. Amino acids in the helix structure were more conserved than those in the loop and turn structure, and the most conserved region was helix3, in which ten highly conserved amino acids were contained, such as I, N, Y, and F. In short, the HB domain was still highly conserved in both *B. napus* and its diploid progenitors.

### Chromosomal localization and orthologous gene analysis of *WOX* genes

**Fig. 4:**
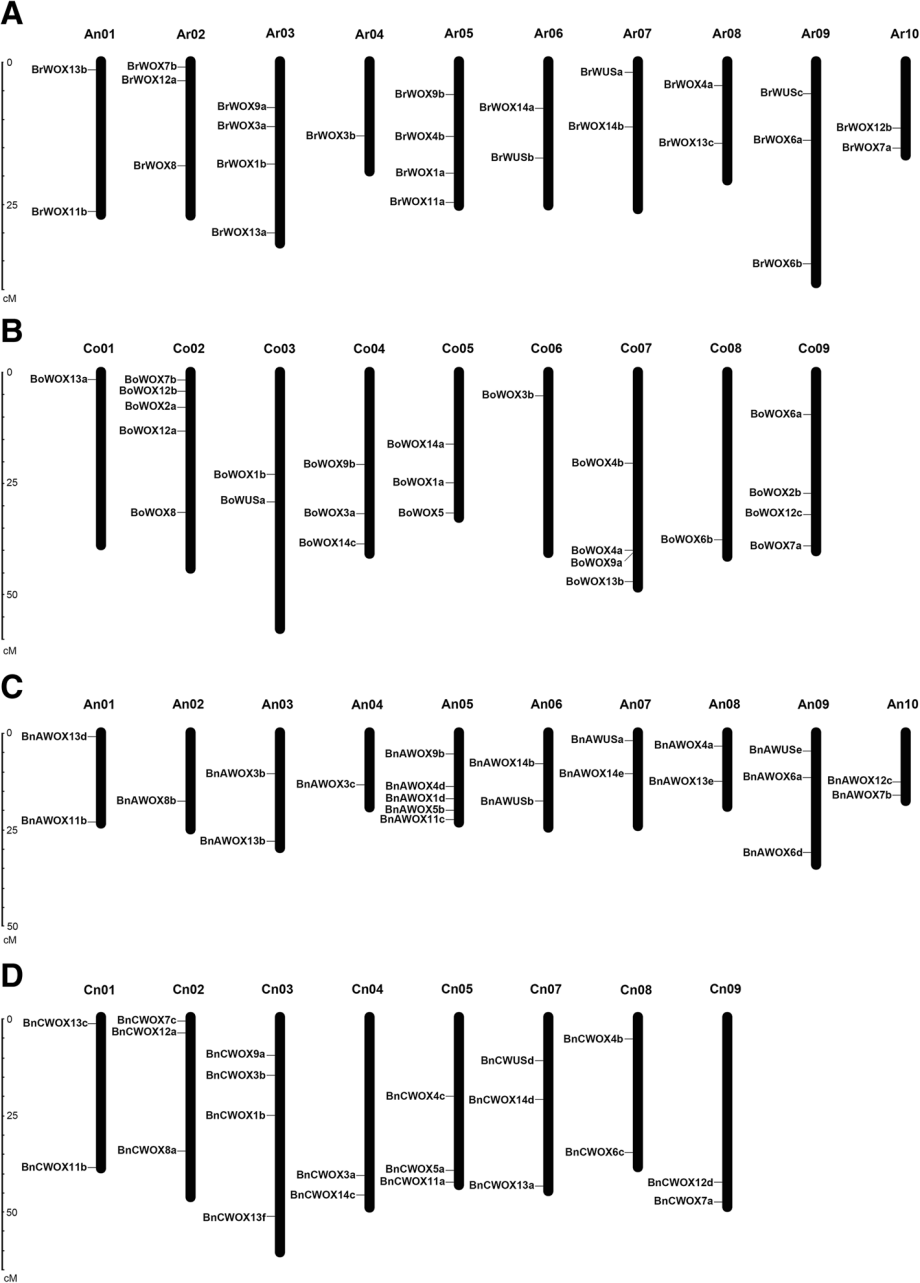
Chromosome distribution of *WOXs* in *B. rapa* (**a**), *B. oleracea* (**b**) and *B. napus* (**c** & **d**). Partial *WOX* genes in *B. oleracea* and *B. napus* located in unassembled scaffolds and these genes were not shown in this figure. The number of chromosomes was indicated at the top of each chromosome. The scale on the left is in megabases (Mb)

The positions of the identified *WOX* genes were drafted to chromosomes by using MapInspector software. Ultimately, 25 *WOX* genes were located on 10 chromosomes in *B. rapa* (Fig. 4a). Evidently, there is only one *WOX* gene on chromosome A_r_04, three on A_r_02 and A_r_09, four on A_r_03 and A_r_05, and two genes on each of the remaining five chromosomes. Twenty-four *WOX* genes were located on nine chromosomes in *B. oleracea*, and the other five genes were located on different scaffolds because they had not been assembled into chromosomes (Fig. 4b). Five genes were distributed in chromosome C_o_02, but in contrast, only one gene was in C_o_01, C_o_06 and C_o_08. Forty-three *WOX* genes were located on 18 instead of 19 chromosomes in *B. napus*, and the other nine genes were located on scaffolds (Fig. 4c). It is worth mentioning that no single gene was located on chromosome C_n_06 in *B. napus*. Comparison of the gene distribution of *B. napus* with *B. rapa* and *B. oleracea* showed the important result that many *WOX* genes retained their relative position in A_r_ and A_n_, but in contrast, only a few genes retained their relative position in C_o_ and C_n_ during the formation of *B. napus*. For example, each pair of chromosomes contained *WOX* genes with the same number and same location, such as A_r_01-A_n_01, A_r_04-A_r_04, A_r_06-A_r_06, A_r_07-A_r_07 and A_r_08-A_r_08, and other chromosome pairs contained *WOX* genes with different numbers but similar locations. However, only one chromosome pair, C_o_02-C_n_02, contained *WOX* genes with the same number and same location. Statistical analysis shows that 21 out of 25 *WOX* genes (84%) were positioned on the assembled chromosomes in *B. rapa*, while 12 out of 24 (50%) in *B. oleracea* maintained their relative position during the formation of *B. napus*. In combination with previous studies, there are two possible reasons for this result. One possibility is that the C_n_ subgenome had more abundant TEs than the A_n_ subgenome [27]. The presence of TEs in the genome could cause the rearrangement of chromosomal sequences, which affects the genomic structure, such as deletion, inversion, and translocation [30]. The other possibility is that the C_n_ subgenome underwent more active homologous exchanges (HEs) than the A_n_ subgenome during polyploidization [27]. HEs refers to the replacement of some chromosomal regions with duplicated copies of the corresponding fragments of the homologous subgenome [31], and this event was found to occur frequently between the two subgenomes of *B. napus* during the hybridization and polyploidization process [27].

### Synteny and duplicated gene analysis of *WOX* genes

**Fig. 5:**
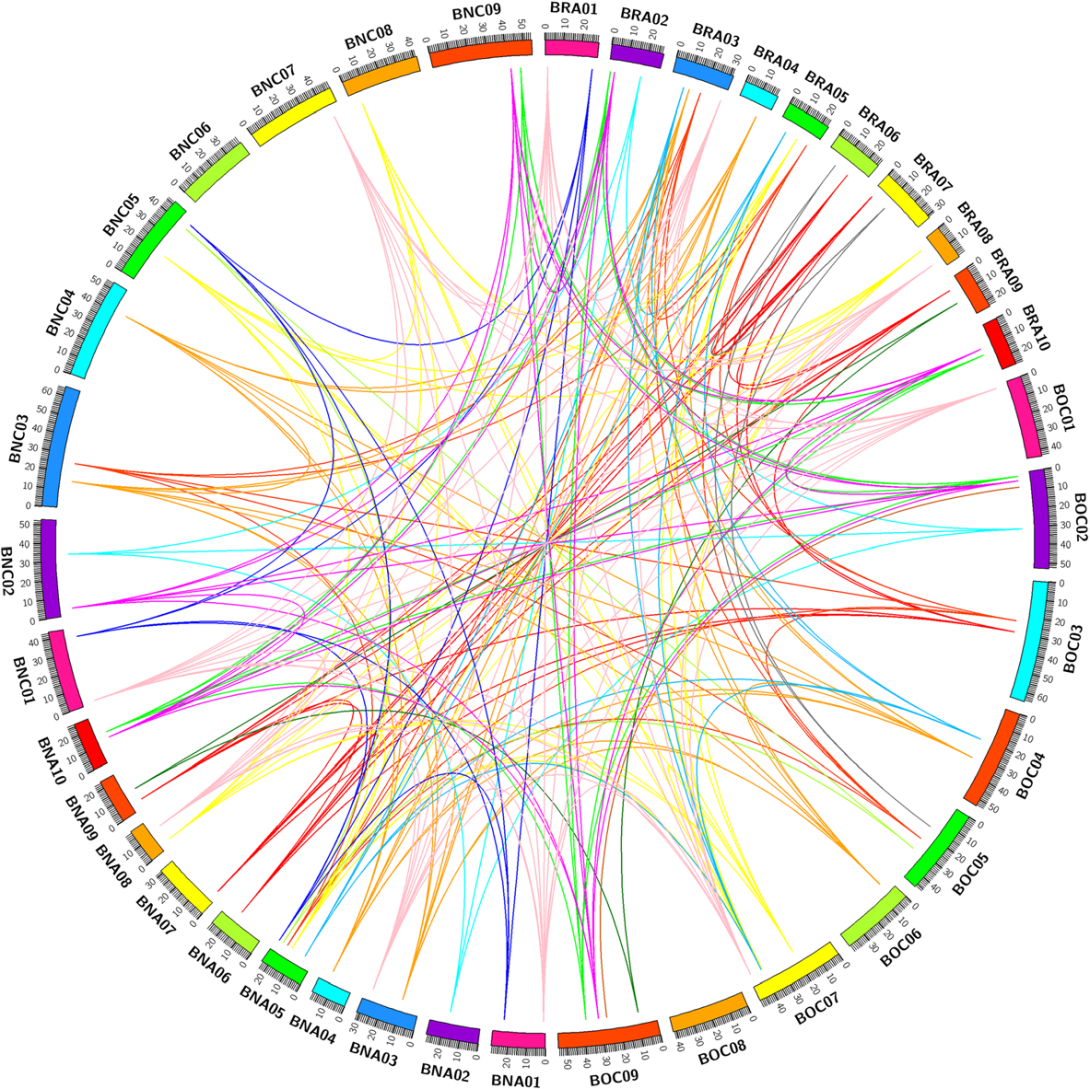
Genome-wide synteny analysis for *WOX* genes among *B. rapa*, *B. oleracea*, *B. napus* and Arabidopsis. BRA01 to BRA10 represented the ten chromosomes in *B. rapa*. BOC01 to BOC09 represented the nine chromosomes in *B. oleracea*. BNA01 to BNA10 and BNC01 to BNC09 represented the ten and nine chromosomes in the A_n_ and C_n_ subgenomes in *B. napus*, respectively. The orthologous and paralogous *WOX* genes were mapped onto the chromosomes and linked by each other. Different colored lines in the inner part represent different kinds of *WOX* genes

Synteny analysis of *WOX* genes in *B. napus* and its diploid progenitors was performed to visualize the locus relationship of homologous *WOX* genes among two genomes (A_r_ & C_o_) and two subgenomes (A_n_ & C_n_). As shown in Fig. 5, two genes linked to each other by one line were syntenic genes, and genes linked by lines of the same color represented the same kind of *WOX* gene, such as *WOX1* and *WOX2*. Thus, we can see that many chromosomes in all four genomes/subgenomes (A_r_, C_o_, A_n_ and C_n_) were connected by the same colored line, which indicated that these genomes/subgenomes were evolutionarily related and the *WOX* genes were so important that most of them were preserved during polyploidization. In addition, *WOX* genes were evenly distributed in these four genomes/subgenomes (Fig. 5). Moreover, the synteny analysis indicated that the syntenic *WOX* gene pairs were widely distributed on the genomes of *B. napus* and its diploid progenitors.

Moreover, to explore whether Darwinian positive selection affected the evolution of the *WOX* genes in *B. napus* and its diploid progenitors, BLASTn [32] and syntenic gene search in BRAD database [33] were used to identify duplicated genes among them. As a result, 13, 10 and 38 segmental duplicated *WOX* gene pairs in the *B. rapa, B. oleracea* and *B. napus* genomes were found respectively. Then, the nonsynonymous (Ka), synonymous (Ks) and Ka/Ks ratios were calculated to estimate the selection pressure among duplicated *WOX* gene pairs. Ka/Ks = 1 means that genes were undergoing a neutral evolutionary process; Ka/Ks > 1 or Ka/Ks < 1 indicate that genes were selected positively or undergoing purified selection, respectively [34]. The Ka/Ks values of all duplicated *WOX* gene pairs in *B. napus* and its diploid progenitors were below one (Additional file 2: Table S2), except one duplicated gene pair (*BnAWOX11b* & *BnCWOX11b*) had no Ka/Ks value in *B. napus* because these two genes had the same sequence.

### Transposable element analysis of WOX proteins

TEs are widely distributed in the genome, and many transposons are located near the host genes [35]. To investigate whether TEs were involved in the expansion of the *WOX* gene family, we identified the TEs located 2000 bp upstream and downstream of the *WOX* genes using the homolog search method [5]. Compared to the TEs near the *WOX* gene family in cotton [5], there were more TEs in both *B. napus* and its diploid progenitors (Table 2). After analysis, 402, 202 and 235 TEs were found in *B. napus*, *B. rapa* and *B. oleracea*, respectively. Thus, a conclusion can be drawn that as early as the formation of the diploid progenitors of *B. napus*, the *WOX* gene family has undergone significant expansion due to the presence of abundant TEs. The three most abundant types of TEs in order of abundance are DNA transposon, LTR retrotransposon and non-LTR retrotransposon. Two types of TEs, Ginger/TDD and R1, were located downstream of *BnAWOX3b* and *BnCWOX12a*, respectively, but these two TEs were not detected near the *WOX* genes in the diploid progenitors. As shown in Table 2, there are 21 kinds of DNA transposons near the *WOX* genes. The most abundant ones are EnSpm/CACTA, MuDR, hAT, Helitron, Mariner/Tc1 and Harbinger. LTR retrotransposons near the *WOX* genes mainly contained four types, Gypsy, Copia, BEL and DIRS. Furthermore, there were 18 kinds of non-LTR retrotransposons near the *WOX* genes of selected species, and the most abundant kind was the L1 type. Statistical analysis shows that 23, 14 and 18 L1-type transposons were found near the *WOX* genes of *B. napus*, *B. rapa* and *B. oleracea*, respectively, and the number of L1-type transposons was much higher than the number of other non-LTR retrotransposons in these species. Compared to TEs, simple repeats were less abundant in *B. napus* and its diploid progenitors. Specifically, there were two simple repeats in *B. napus*, but only one in *B. rapa* and none in *B. oleracea*.

**Table 2:** The TEs around *WOX* genes locus

Repeat Class	No. of elements in *B. napus*	No. of elements in *B. rapa*	No. of elements in *B. oleracea*
Integrated Virus	6	2	4
Caulimoviridae	6	2	4
Interspersed Repeat	1	1	0
DNA transposon	203	96	118
Academ	1	0	1
Crypton	2	1	1
Dada	1	1	2
EnSpm/CACTA	40	18	20
Ginger1	2	0	3
Ginger2/TDD	1	0	0
Harbinger	15	10	12
Helitron	23	6	12
IS3EU	2	2	0
ISL2EU	0	1	0
Kolobok	5	0	1
Mariner/Tc1	18	7	12
Merlin	1	1	0
MuDR	29	15	13
P	3	5	3
Polinton	9	5	4
Sola	1	1	2
Sola2	1	1	1
Sola3	0	0	1
Zisupton	0	0	1
Transib	1	1	0
hAT	25	13	20
piggyBac	2	1	1
Endogenous Retrovirus	5	3	4
ERV1	1	1	0
ERV2	1	1	1
ERV3	1	1	1
LTR Retrotransposon	114	56	57
BEL	6	2	2
Copia	35	13	14
DIRS	0	1	1
Gypsy	68	37	34
Non-LTR Retrotransposon	73	45	52
CR1	6	4	6
Crack	2	0	1
Daphne	3	1	3
I	2	0	2
Jockey	3	1	1
L1	23	14	18
L2	1	0	2
NeSL	0	2	0
Nimb	1	1	0
Outcast	1	1	0
Penelope	9	5	3
R1	1	0	0
RTE	3	1	5
RTEX	3	1	0
Rex1	1	1	0
SINE	8	5	4
SINE2/tRNA	8	5	4
Tad1	0	2	2
Tx1	4	2	3
Simple Repeat	2	1	0
Satellite	2	1	0
SAT	2	1	0
Transposable Element	402	202	235
Total	411	206	239

### Gene expression pattern analysis of *WOX* genes

**Fig. 6:**
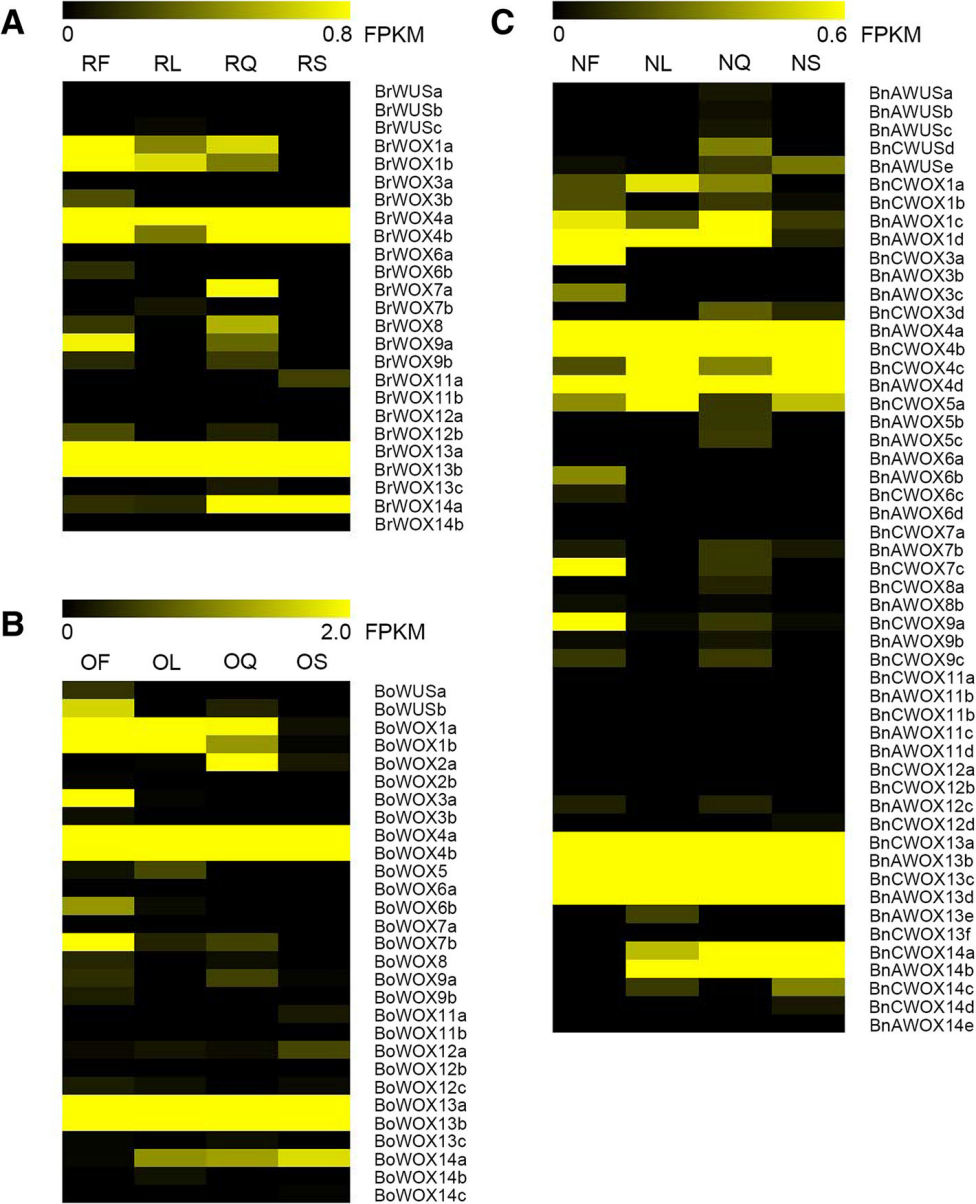
Expression patterns of *WOXs* in four major tissues of *B. napus* and its diploid progenitors. **a** The expression pattern of *WOXs* in *B. rapa*. **b** The expression pattern of *WOXs* in *B. oleracea*. **c** The expression pattern of *WOXs* in *B. napus*. The scale is FPKM

To gain insights into the putative biological functions of *WOX* genes, we investigated their expression patterns in four tissues (leaves, stems, flowers and siliques) of *B. napus* and its two diploid progenitors based on our RNA-seq data (Additional file 3: Table S3). As shown in Fig. 6, we found that *WOX* genes are widely expressed in these four tissues, suggesting that *WOX* genes have multiple biological functions and operate in different tissues. In addition, the expression of *WOX* genes from different clades had different characteristics, and the specific characteristics were as follows: The genes of the intermediate clade were generally not expressed in all tissues, except *BrWOX9a* and *BnCWOX9a*, which were expressed in flowers; In the ancient clade, most of the *WOX13* homologous genes were widely expressed in the four tissues and had a high expression level, and *WOX14* homologous genes were not expressed in flowers; In the WUS clade, only the homologous genes of *WOX4* were widely expressed in the four tissues. *WOX3* and *WOX6* homologous genes were not significantly expressed in all four tissues except *BnCWOX3a*, *BoWOX3a*, *BnAWOX6a* and *BoWOX6b*, which were detected in flowers. In addition, the homologous genes of *WOX1* generally were not expressed in stems of *B. napus* or its two diploid progenitors. In brief, the most active genes were the genes in the ancient clade; conversely, the least active genes were those in the intermediate clade in *B. napus* and its two diploid progenitors, which suggested that *WOX* genes in the ancient clade play important roles in the process of growth and development of *B. napus* and its two diploid progenitors.

To explore whether the expression pattern of the *WOX* genes in the four tissues changed in allotetraploid *B. napus* and its two diploid progenitors, we selected the previous 33 pairs of genes that may have evolutionary relationships for analysis. As shown in Table 3, the fragments per kilobase of exon per million reads mapped (FPKM) values of the 33 gene pairs with potential direct evolutionary relationships were collected. As a result, we found that there was no direct relationship between the same gene structure and the same expression trend in these gene pairs. For example, 13 out of the 23 (approximately 56%) gene pairs with the same gene structure had absolutely different expression trends in the four tissues, such as *BoWOX13a* & *BnCWOX13c* and *BoWOX4a* & *BnCWOX4b*. However, 4 out of 10 (40%) gene pairs with distinct gene structures had the same expression trend, such as *BoWOX12b* & *BnCWOX12a* and *BrWOX11b* & *BnAWOX11d*. These results suggested that many *WOX* genes have no obvious changes at the DNA level, but most of the genes presented different characteristics at the expression level, which might be caused by the changes in gene expression regulation in the process of polyploidization.

**Table 3:** The expression patterns of *WOX* gene pairs with potential direct evolutionary relationships

Clade	Gene pairs with evolutionary relationship^a^	Flowers_FPKM	Leaves_FPKM	Siliques_FPKM	Stems_FPKM	Gene structure^b^
Ancient clade	*BoWOX14c*	0	0	0	0.04	D
	*BnCWOX14c*	0	0.14	0	0.31	
	*BrWOX14b*	0	0	0	0	S
	*BnAWOX14e*	0	0	0	0	
	*BrWOX14a*	0.15	0.13	2.85	1.42	S
	*BnAWOX14b*	0	0.87	1.05	0.83	
	*BoWOX14a*	0.06	1.15	1.26	1.74	S
	*BnCWOX14a*	0	0.44	2.60	1.92	
	*BrWOX13c*	0	0	0.09	0	D
	*BnCWOX13f*	0	0	0	0	
	*BoWOX13a*	21.94	24.69	53.65	40.50	S
	*BnCWOX13c*	3.90	14.43	8.26	14.86	
	*BrWOX13a*	7.20	4.19	2.34	5.96	S
	*BnAWOX13b*	4.03	8.51	3.32	11.46	
Intermediate clade	*BrWOX9b*	0.14	0	0.19	0	S
	*BnAWOX9b*	0.03	0	0.05	0	
	*BoWOX9b*	0.24	0	0	0	S
	*BnCWOX9c*	0.13	0	0.14	0	
	*BoWOX9a*	0.37	0	0.51	0.06	D
	*BnCWOX9a*	1.63	0.03	0.13	0.03	
	*BrWOX8*	0.18	0	0.56	0	D
	*BnAWOX8b*	0.04	0	0.02	0	
	*BoWOX8*	0.30	0	0.12	0	S
	*BnCWOX8a*	0	0	0.09	0	
	*BrWOX12b*	0.24	0	0.11	0	S
	*BnAWOX12c*	0.08	0	0.08	0	
	*BoWOX12c*	0.22	0.14	0	0.09	S
	*BnCWOX12d*	0	0	0	0.04	
	*BoWOX12b*	0	0	0	0	D
	*BnCWOX12a*	0	0	0	0	
	*BoWOX12a*	0.09	0.17	0.11	0.56	S
	*BnCWOX12b*	0	0	0	0	
	*BrWOX11a*	0	0	0	0.21	D
	*BnAWOX11c*	0	0	0	0	
	*BrWOX11b*	0	0	0	0	D
	*BnAWOX11d*	0	0	0	0	
WUS clade	*BrWOX4a*	11.13	1.17	33.37	43.02	S
	*BnAWOX4a*	2.13	1.13	8.17	19.94	
	*BoWOX4a*	23.56	5.15	46.29	11.42	S
	*BnCWOX4b*	4.08	1.65	16.69	30.36	
	*BrWOX4b*	0.95	0.38	1.62	6.57	S
	*BnAWOX4d*	0.79	0.94	1.55	16.73	
	*BoWOX4b*	2.98	6.36	5.71	10.23	S
	*BnCWOX4c*	0.19	0.68	0.31	3.33	
	*BrWOX3a*	0	0	0	0	S
	*BnAWOX3b*	0	0	0	0	
	*BoWOX3b*	0.12	0	0	0	S
	*BnCWOX3d*	0	0	0.22	0.10	
	*BrWOX3b*	0.25	0	0	0	S
	*BnAWOX3c*	0.31	0	0	0	
	*BoWOX3a*	2.01	0.05	0	0	S
	*BnCWOX3a*	0.60	0	0	0	
	*BoWOX6a*	0	0	0	0	D
	*BnAWOX6a*	0	0	0	0	
	*BrWOX6b*	0.15	0	0	0	D
	*BnCWOX6c*	0.08	0	0	0	
	*BrWUSc*	0	0.03	0	0	D
	*BnAWUSe*	0.04	0	0.14	0.28	
	*BoWUSa*	0.41	0	0	0	S
	*BnAWUSb*	0	0	0.04	0	
	*BoWOX5*	0.15	0.58	0	0	S
	*BnCWOX5a*	0.33	0.63	0.13	0.45	
	*BrWOX7a*	0	0	0.79	0	S
	*BnAWOX7b*	0.07	0	0.13	0.06	
	*BoWOX7b*	4.48	0.28	0.52	0	S
	*BnCWOX7c*	1.14	0	0.13	0	

^a^ Gene pairs with evolutionary relationship refer to two genes derived from tetraploid and one of its two diploid progenitors respectively, and which are at the same last-level branch in the phylogenetic tree and with the closest evolutionary distance

^b^ Gene structure is to show whether the gene pair has the same intron/exon structure, the same is represented by S, and the difference is represented by D

### Bias expression analysis of *WOX* genes

To explore the expression bias of *WOX* genes in allotetraploid *B. napus* in different tissues, bias analysis was performed based on FPKM. The 33 previously selected *WOX* gene pairs could be grouped according to homology, so there were 12 groups of *WOX* genes, such as *WOX14*, *WOX3* and *WUS*. Since two groups of genes (*WOX11* & *WOX5*) were present in only one diploid progenitor, the bias analysis cannot be performed on these genes. Therefore, we selected the remaining 10 groups of *WOX* genes for bias analysis.

The expression bias of the *WOX* genes showed different characteristics in different tissues. As shown in Additional file 4: Table S4, in flowers, the expression of 9 groups of *WOX* genes were biased towards *B. rapa*, and only the expression of *WOX12* was biased towards *B. oleracea*. In leaves, the expression of 7 groups of *WOX* genes was biased towards *B. rapa*, and only the expression of *WOX14* was biased towards *B. oleracea*; additionally, the expression of two other groups of genes (*WOX6* & *WOX8*) had no obvious bias. In stems, the expression of 6 groups of *WOX* genes was biased towards *B. rapa*, and the expression of two groups of genes (*WOX4* & *WOX14*) was biased towards *B. oleracea*; additionally, two groups of genes (*WOX6* & *WOX8*) had no obvious bias. In siliques, the expression of 4 groups of *WOX* genes was biased towards *B. rapa*, while 5 groups of *WOX* genes were biased towards *B. oleracea*, and only *WOX6* had no obvious bias.

Hence, the expression bias of *WOX* genes in stems and leaves was largely identical to each other, except that the expression of *WOX4* was biased towards *B. rapa* in leaves, while biased towards *B. oleracea* in stems. The expression of *WOX6* had no bias in stems, leaves and siliques but was biased towards *B. rapa* in flowers. The expression of *WOX14* was biased towards *B. oleracea* in stems, leaves and siliques. Three groups of genes (*WUS*, *WOX3* and *WOX13*) were biased towards *B. rapa* in all four tissues. In general, the results showed that the expression of *WOX* genes in *B. napus* was biased towards *B. rapa* in stems, leaves and flowers, while they had no obvious bias in siliques.

## Discussion

As an important gene family in plants, *WOX* genes encode WOX proteins to regulate cell division and differentiation, thereby influencing plant growth and development [8–10]. Previous studies on the *WOX* gene family have been performed in many plants, including cotton [5], tabacco [6], maize [7], watermelon [36]. A recent study just analyzed the *WOX* gene family and their stress- and hormone-responsive patterns in *B. napus* [37], our study focused more on the changes in *WOX* genes during the polyploidization process. Compared to their study [37], less but more accurate *WOX* genes were identified in *B. napus* and its diploid progenitors in current study. In addition, *B. rapa*, *B. oleracea* and *B. napus* were a nice group to explore polyploidization-related issues in *Brassica*, but current studies on polyploidization mainly focused on comparative analysis of transcriptomes [38]. Thus, it was necessary to identify the *WOX* gene family completely, analyze the changes in this gene family during polyploidization and provide a reference at the genome level for follow-up polyploidization studies. Hence, we identified and analyzed the *WOX* gene family in *B. napus* and its diploid progenitors (*B. rapa* and *B. oleracea*) in this study, with the aim of understanding the evolution of this gene family during the natural polyploidization of allotetraploid *B. napus*.

### The *WOX* gene family expanded in *B. napus*

The allotetraploid species *B. napus* is an important global oil crop producing biofuels and industrial compounds [39]. *B. napus* was formed ~ 7500 years ago by allopolyploidy, a process in which hybridization happened between ancestors *B. rapa* (Asian cabbage or turnip) and *B. oleracea* (Mediterranean cabbage), and then the chromosomes were doubled [27].

In the present study, a total of 52 *WOX* genes were identified in *B. napus*, which is the largest number reported in plants to date [5–7, 40]. The quantitative advantage demonstrated that the *WOX* gene family of *B. napus* has undergone significant expansion during the formation of this species. Each Brassicaceae genome underwent a whole-genome duplication (WGD) event ~ 35 MYA ago [41, 42]. Additionally, after their divergence from the Arabidopsis lineage, the *Brassica* genome experienced a *Brassica*-lineage-specific WGT event ~ 15.9 MYA [26, 43]. Therefore, it is apparent that the *Brassica* genome underwent paleopolyploidization [42, 43]. Polyploidization is the leading cause of the increase in the number of *WOX* genes. The polyploidization is a significant event in the evolution of flowering plants, which may play an important part in the adaptation of plants to new living environments [44]. In addition, segmental or tandem duplication also gives rise to an increase in the number of genes [5, 45, 46]. Segmented duplication often occurs in plants because most plants have undergone polyploidization events and thus retain a large number of duplicated chromosomal blocks in their genome [45]. For example, segmental duplication is the primary force for *Hsf* gene expansion in sesame [47]. In our study, 30 out of 52 *WOX* genes were involved in segmental duplication, that is, approximately 58% of the *WOX* genes have experienced segmental duplication events, suggesting that this event played an important role in the expansion of the *WOX* gene family. Tandem duplicated genes were defined as an array of at least two homologous genes within 100 kb [48]. Tandem duplication provides a means to amplify important adaptive resistance genes [46]. Unfortunately, we did not find any tandem duplicated genes in this study, but it is undeniable that it is still a basic factor for gene expansion. Additionally, abundant TEs can also lead to the expansion of the *WOX* gene family. Studies have shown that the proliferation of TEs leads to genome expansion in Corydoradinae catfishes [49]. In this study, 402, 202 and 235 TEs were found near *WOX* genes in *B. napus*, *B. rapa* and *B. oleracea*, respectively. Compared with a previous study [5], the numbers of TEs were very large, so the conclusion can be drawn that the *WOX* gene family has undergone significant expansion due to the presence of abundant TEs. After gene duplication, the new gene would be redundant with the pre-existing gene, and this redundancy has been considered an essential driving force in the process of evolutionary innovation [46]. At present, some models can be used to understand gene duplication events, such as neofunctionalization and DDC subfunctionalization, which provide a novel theoretical framework for further study of this process [46].

### Gene loss events occurred in the *WOX* gene family of *B. napus*

Gene loss always occurs when the genomic sequence rearranges after hybridization and chromosome doubling [50]. Although the number of *WOX* genes in *B. napus* is very large, gene loss also occurred during polyploidization, which was determined from the comparison of the number of *WOX* genes in *B. napus* (52) with the sum of the gene numbers in its two progenitors (54).

*B. napus*, a typical allotetraploid, is an ideal model to study natural polyploidy [27]. The ancestor of *Brassica* underwent a WGT event after separation from Arabidopsis, so theoretically, the genes in Arabidopsis should have three homologous genes in diploid *Brassica*. However, in this study, only two of 15 and three of 15 *AtWOX* genes had three orthologous genes in the *B. rapa* and *B. oleracea* genomes, respectively. Furthermore, nine of 15 *AtWOX* genes had two orthologous genes in both the *B. rapa* and *B. oleracea* genomes. The remaining genes had zero or one orthologous gene in the *B. rapa* and *B. oleracea* genomes. Hence, the conclusion can be drawn that most *WOX* genes experienced gene loss after the noted WGT event in the formation of *B. rapa* and *B. oleracea*. Furthermore, *B. napus* was formed by natural hybridization and polyploidization of *B. rapa* and *B. oleracea*. Fourteen of fifteen *AtWOX* genes had less than six orthologous genes in the *B. napus* genome. However, only one *AtWOX* gene had six orthologous genes in *B. napus*, and these six genes were evenly distributed in the A_n_ and C_n_ subgenomes, which indicated that most *WOX* genes also experienced gene loss during the formation of *B. napus*.

Although gene loss events have happened in *B. napus* and its diploid progenitors, *B. napus* still had more *WOX* genes in its genome compared to the allotetraploid *Nicotiana tabacum* [6] and *Gossypium hirsutum* [5].

### The *WOX* gene family is highly conserved at the DNA and protein level in *B. napus*

WOX proteins belong to a plant-specific branch in the superfamily of HB transcription factors [2]. Previous research has found that the HB domain may have already diverged before the separation of animals, plants and fungi [51]. The HB domain of the WOX proteins contains one loop, one turn and three helix structures [5]. It has been reported that the HB domain of the *WOX* genes contains some highly conserved amino acids in these three helix structures; for instance, it contains Q, L and E in helix 1, P, I and L in helix 2, and I, N, V, F, Y, W, F, Q, N and R in helix 3 [28]. Moreover, previous studies have demonstrated that the amino acids in the loop and turn structure are less conserved [5]. Evidently, our results regarding the HB domain of the WOX proteins in *B. napus* and its diploid progenitors were consistent with the above conclusions. According to previous studies, *WOX* genes can be divided into three clades, namely, the ancient clade, intermediate clade and WUS clade [5, 6], as was observed in our present study (Fig. 1).

After analysis, *WOX* genes, whether in allotetraploid *B. napus* or its diploid progenitors, were highly conserved at the DNA and protein levels, including the gene structure of *WOX* genes, conserved amino acids in the HB domain, and types of motifs. Specifically, it was calculated that at least 70% of *WOX* genes maintained the same gene structure during the formation of allotetraploid *B. napus*. Furthermore, according to our statistical analysis, 67% of *WOX* genes maintained their relative positions on the chromosomes during evolution. In summary, *WOX* genes were highly conserved at the DNA level during the polyploidization process from diploid to allotetraploid *B. napus*.

It is generally believed that *B. napus* (2n = 4x = 38, A_n_C_n_) was reunited by the hybridization of an A-genome material *B. rapa* (2n = 20, A_r_) with a C-genome material *B. oleracea* (2n = 18, C_o_), followed by a chromosome doubling event [27]. Our study showed that the distribution features of the *WOX* genes on four genomes/subgenomes (A_r_, C_o_, A_n_ and C_n_) were roughly consistent with the above hypothesis, which confirmed that *WOX* genes in *B. napus* were highly conserved during hybridization and polyploidization.

### The expression features of the *WOX* gene family were changed in *B. napus* compared with its diploid progenitors

A previous study has shown that some *WOX* genes are expressed at low levels in reproductive tissues and are not expressed in their vegetative tissues in cotton [5]. In our study, most of the *WOX* genes were also expressed at low levels or even were not expressed in the four tested tissues. The reason for this might be that the *WOX* genes are often expressed at some restricted locations, such as embryos or quiescent centers in roots. Segmental duplication is an important way to increase diversity at the DNA level. After duplication, genes may still function as before or may acquire only part of the function of the previous gene, namely, subfunctionalization; moreover, it is also possible for genes to obtain new functions, namely, neofunctionalization [52]. In *B. napus*, the differential expression pattern of these duplicated genes indicated that they might have undergone functional divergence after duplication. For example, the expression pattern of *BnCWOX13e*, which is the duplicated gene of *BnCWOX13a*, is completely different from that of *BnCWOX13a*, which indicated that *BnCWOX13e* might only have acquired some of the functions of the original gene or might have acquired new functions. Of course, there are still some duplicated gene pairs that maintain the previous expression patterns, such as *BnAWOX4b* & *BnCWOX4b*, and *BnCWOX3a* & *BnAWOX3c*. These genes may have maintained their previous functions.

Furthermore, the expression characteristics of some *WOX* genes suggested that their function is consistent with that of homologous genes in Arabidopsis. For example, studies have shown that WOX6 regulates the development of ovules [18], and the homologs of *WOX6* expressed only in reproductive tissue (flowers) in our data, indicating they may play similar roles in *B. napus* and its two diploid progenitors; however, this speculation needs to be tested experimentally. Similarly, *AtWOX14* was mainly involved in the lignification process in Arabidopsis [24], and we noticed that the homologs of *WOX14* were not expressed in flowers, so it is speculated that *WOX14* homologous genes also have a similar function to *AtWOX14*. Moreover, an interesting conclusion was found in our study, which was that there was no significant correlation between gene structure and expression pattern. Specifically, approximately 56% of the gene pairs in *B. napus* and its diploid progenitors, which were conserved at the DNA level, were changed at the expression level.

In short, our study showed that approximately 70% of the members of the *WOX* gene family in *B. napus* maintained their gene structure during the polyploidization process, but approximately 56% of them changed significantly at the expression level, revealing that the *WOX* gene family has changed during polyploidization.

## Conclusions

In this study, 52, 25 and 29 *WOX* genes were identified in allotetraploid *B. napus*, the A_n_ genome donor *B. rapa* and the C_n_ genome donor *B. oleracea*, respectively. After analysis, whole genome duplication, segmental duplication and abundant TEs were determined to be the three major impetuses for the expansion of the *WOX* gene family during the process of polyploidization. Moreover, gene loss events have happened in the *WOX* gene family in *B. napus* during polyploidization. Additionally, the *WOX* gene family in *B. napus* was highly conserved at the DNA and protein level but changed at the expression level during polyploidization. Together, these results can increase our understanding of the evolution of the *WOX* gene family and provide a reference for future polyploidization analysis.

## Methods

### Plant materials and transcriptome sequencing

The seeds of *B. napus* (cv. Darmor), *B. rapa* (cv. Chiifu) and *B. oleracea* (cv. Jinzaosheng) were obtained from the Oil Crops Research Institute, Chinese Academy of Agricultural Sciences. These materials were grown in soil under natural conditions (Wuhan, China). Some well-grown inflorescences were bagged to prevent pollen contamination before flowering. Young leaves, inflorescence stems, blooming flowers and siliques (10 DAP) from 6-month-old plants were collected and frozen immediately in liquid nitrogen for transcriptome sequencing. The platform for transcriptome sequencing was Illumina (HiSeq X-Ten).

### Identification of *WOX* genes

The *WOX* genes in allotetraploid *B. napus* and its diploid progenitors (*B. rapa* and *B. oleracea*) were comprehensively identified by three methods. The first method is the BLASTp search method. Fifteen protein sequences encoded by *WOX* genes in *A. thaliana* were acquired from the TAIR database (http://www.arabidopsis.org/), which were used as query sequences to perform the BLASTp search (E-value <1e-5) with all protein sequences of the three selected *Brassica* species, using the BRAD database (http://brassicadB.org/brad/) [28]. Then, the repeated genes were removed manually, and original candidate genes were obtained. The second method is searching syntenic genes in the BRAD database by inputting the 15 *WOX* gene IDs of *A. thaliana*. The third method is used to screen the candidate genes accurately by searching for the conserved HB domain of the corresponding proteins. Three public databases, including NCBI Conserved Domain Database (CDD; https://www.ncbi.nlm.nih.gov/cdd) [53], SMART database (http://smart.embl-heidelberg.de/) [54], and Pfam database (http://pfam.xfam.org/) [55], were used to search the HB domain of candidate sequences, and the domain ID is PF00046, SM000389 and PF00046 in each database, respectively. Sequences not containing the complete conserved HB domain were removed. Finally, all *WOX* genes were identified in *B. napus* and its diploid progenitors in currently available data, and the identified genes were named according to the homologous relationship with *WOX* genes in *A. thaliana*. The coding sequences of the identified *WOX* genes and the amino acid sequences of the corresponding WOX proteins in the three selected *Brassica* species were acquired from the BRAD database. *WOX* genes of rice were identified using the first and the last methods mentioned above, and all information regarding rice was taken from the open rice database (http://rice.plantbiology.msu.edu/) [56].

### Chromosome location and structure of *WOX* genes

Information about the physical locations of *WOX* genes in the genomes of *B. napus* and its diploid progenitors was collected from the BRAD database, and their positions were drafted to chromosomes by using MapInspector software. The structures of the *WOX* genes were displayed using the gene structure display server 2.0 (GSDS 2.0; http://gsds.cbi.pku.edu.cn//index.php) [57] program to obtain their exon/intron composition information.

### Conserved motifs and HB domain analysis of WOX proteins

The conserved motifs were investigated using the MEME tool (http://meme-suite.org/tools/meme) [58], with the number of found motifs as nine and the other parameters as default values. The location diagram of the HB domain of the *WOX* genes was drawn using IBS 1.0.3 software (http://ibs.biocuckoo.org/download.php) [59], according to the related information from the SMART database. For conserved sequence logo analysis, the conserved HB domain sequences of *WOX* genes from *B. rapa*, *B. oleracea*, *B. napus* and Arabidopsis were aligned by ClustalX 2.1 [60], and the multiple alignment results were submitted to an online tool, WEBLOGO (http://weblogo.berkeley.edu/logo.cgi) [61], for generating the logos.

### Characteristics and phylogenetic relationship analysis of WOX proteins

The WOX protein sequences in *B. napus* and its diploid progenitors were analyzed for physical and chemical characteristics by the online ProtParam tool of ExPASy (http://weB.expasy.org/protparam/) [62], including the number of amino acids, MW, theoretical pI, II, and GRAVY. The full-length WOX proteins in *B. rapa*, *B. oleracea*, *B. napus*, *A. thaliana* and rice were aligned using ClustalW. Phylogenetic analysis of WOX proteins was performed using MEGA 7.0.26 [63] with the neighbor-joining (NJ) method based on the Poisson model, and the bootstrap method was used to test the tree with 1000 replicates. An online website iTOL (Interactive Tree of Life, http://itol.embl.de/) [64] was used to annotate the tree.

### Duplications and syntenic analysis of *WOX* genes

Two methods were used to identify the duplicated *WOX* genes in *B. napus* and its diploid progenitors. First, the BLASTn program was used with both coverage and identity > 80% were considered as candidate duplicated genes [32]. Second, gene IDs were inputted into the BRAD database to search for their syntenic genes [33]. The common duplicated genes identified by the two methods were considered as the final duplicated genes. DNAsp5 software [65] was applied to calculate the Ks, Ka and evolutionary constraint (Ka/Ks) between the duplicated *WOX* gene pairs. Then, Ks values were used to estimate the divergence events, and the divergence time of duplicated genes was calculated using the formula T = Ks/2r Mya (Millions of years), where ‘r’ is equal to 1.5 × 10^− 8^ according to a previous study [66]. The syntenic genes of *WOX* genes in *B. napus* and its diploid progenitors were searched on the BRAD database, and then Circos software [67] was used to show the syntenic relationship between them.

### Analysis of transposable elements near WOX genes

Methods for detecting TEs include de novo prediction, the homology-based method, the structure-based method, and the comparative genomic method, among which the most common method is the homology-based method [68], which is based on detecting homology to known TEs. To understand whether TEs play roles in the expansion of the *WOX* gene family, we detected TEs 2000 bp upstream and downstream of *WOX* genes based on the homology-based method [5]. TEs were identified using the Repeat Masking tool in the known TEs database Repbase (https://www.girinst.org/censor/index.php) [69, 70]. Finally, we calculated the total number and number of various types of TEs present near *WOX* genes in *B. napus* and its diploid progenitors.

### Transcriptome data analysis and gene expression heatmap of *WOX* genes

The Illumina RNA-seq data were analyzed to reveal the expression patterns of *WOX* genes in *B. napus* and its diploid progenitors. The raw data of RNA-seq reads were deposited in the NCBI database under accession number (SRR7816633-SRR7816668). FPKM values were used to represent the gene expression levels. The heatmap of the expression patterns of *WOX* genes was generated by MutiExperimentViewer (MeV; version 4.8.1) software.

## Additional files

Additional file 1: **Table S1.** The characteristics of *WOX*s in *B. napus* and its diploid progenitors with their Arabidopsis orthologs. (DOCX 32 kb)

Additional file 2: **Table S2.** Estimated Ka/Ks ratios of duplicated *WOX* gene pairs in *B. napus* and its diploid progenitors. (DOCX 25 kb)

Additional file 3: **Table S3.** The FPKM values of all *WOX* genes of *B. napus* and its diploid progenitors (*B. rapa* and *B. oleracea*) in four tissues. (XLSX 21 kb)

Additional file 4: **Table S4.** The |log_2_FC| of *WOX* genes in four tissues. (XLSX 10 kb)

## Abbreviations

*A. thaliana*: *Arabidopsis thaliana*
*B. napus*: *Brassica napus*
*B. oleracea*: *Brassica oleracea*
*B. rapa*: *Brassica rapa*
BLAST: Basic Local Alignment Search Tool
FPKM: Fragments per kilobase million
GA: Gibberellin
GRAVY: Grand average of hydropathicity
HB: Homeobox
HEs: Homologous exchanges
II: Instability index
Ka: Non-synonymous substitution
Ks: Synonymous substitution
MW: Molecular weight
MYA: Million years ago
NJ: Neighbor-joining
TEs: Transposable elements
WGD: Whole-genome duplication
WGT: Whole-genome triplication
*WOX*: *WUSCHEL*-related homeobox
