# Supplementary material for: Genome-wide identification and analysis of the WUSCHEL-related homeobox (WOX) gene family in allotetraploid Brassica napus reveals changes in WOX genes during polyploidization
Source: BMC Genomics. 2019 Apr 25;20:317. doi: 10.1186/s12864-019-5684-3 (PMC6482515; doi:10.1186/s12864-019-5684-3)
Supplement: Supplementary file 1 — Table S1. The characteristics of WOXs in B. napus and its diploid progenitors with their Arabidopsis orthologs. (DOCX 32 kb) [file 12864_2019_5684_MOESM1_ESM.docx]

**Table S1** The characteristics of *WOX*s in *B. napus* and its diploid progenitors with their Arabidopsis orthologs.

| Gene name | BRAD ID | Chromosome | | | Predicated protein information | | | | | Orthologous gene |
| --- | --- | --- | --- | --- | --- | --- | --- | --- | --- | --- |
|  |  | No. | Start | End | MW (kDa) | pI | II | Aliphatic index | GRAVY |  |
| BrWUSa | Bra039894 | A07 | 1811494 | 1813083 | 34278.64 | 6.87 | 51.57 | 48.77 | -1.035 | AT2G17950 |
| BrWUSb | Bra024485 | A06 | 16740714 | 16742385 | 33383.63 | 7.8 | 49.78 | 49.46 | -0.996 | AT2G17950 |
| BrWUSc | Bra037245 | A09 | 5416266 | 5417980 | 35256.37 | 6.83 | 53.72 | 47.4 | -1.084 | AT2G17950 |
| BrWOX1a | Bra022267 | A05 | 19472401 | 19474321 | 40014.79 | 8.39 | 47 | 52.25 | -0.929 | AT3G18010 |
| BrWOX1b | Bra001694 | A03 | 17917095 | 17919123 | 40197.58 | 6.97 | 54.46 | 51.1 | -1.04 | AT3G18010 |
| BrWOX3a | Bra000484 | A03 | 11356689 | 11357728 | 26087.02 | 9.33 | 62.76 | 70.31 | -0.604 | AT2G28610 |
| BrWOX3b | Bra035688 | A04 | 12918967 | 12920054 | 27143.24 | 9.32 | 75.63 | 66.28 | -0.669 | AT2G28610 |
| BrWOX4a | Bra014055 | A08 | 4029047 | 4030092 | 29199.63 | 9.28 | 62 | 58.4 | -0.851 | AT1G46480 |
| BrWOX4b | Bra032212 | A05 | 12987493 | 12988566 | 28555.1 | 9.63 | 70.99 | 56.33 | -0.847 | AT1G46480 |
| BrWOX6a | Bra017448 | A09 | 13657232 | 13658585 | 31872.12 | 5.35 | 54.38 | 67.86 | -0.923 | AT2G01500 |
| BrWOX6b | Bra026791 | A09 | 35463697 | 35464850 | 30660.66 | 5.52 | 74.69 | 51.89 | -1.355 | AT2G01500 |
| BrWOX7a | Bra009132 | A10 | 15115158 | 15115834 | 22073.82 | 8.99 | 47.01 | 64.48 | -0.816 | AT5G05770 |
| BrWOX7b | Bra028749 | A02 | 861290 | 861904 | 20051.41 | 7.76 | 47.59 | 64.43 | -0.844 | AT5G05770 |
| BrWOX8 | Bra022008 | A02 | 18150945 | 18152495 | 34252.71 | 6.23 | 45.19 | 71.14 | -0.473 | AT5G45980 |
| BrWOX9a | Bra022960 | A03 | 7962183 | 7964428 | 43945.47 | 6.62 | 61.04 | 68.93 | -0.552 | AT2G33880 |
| BrWOX9b | Bra005464 | A05 | 5636162 | 5637739 | 43341.87 | 8.35 | 62.33 | 66.51 | -0.557 | AT2G33880 |
| BrWOX11a | Bra031980 | A05 | 24658530 | 24660116 | 34955.04 | 6.29 | 57.19 | 77.48 | -0.249 | AT3G03660 |
| BrWOX11b | Bra036446 | A01 | 26237296 | 26238679 | 29135.19 | 5.97 | 69.97 | 57.4 | -0.526 | AT3G03660 |
| BrWOX12a | Bra023638 | A02 | 3286208 | 3288015 | 30435.49 | 6.09 | 73.86 | 54.29 | -0.663 | AT5G17810 |
| BrWOX12b | Bra002108 | A10 | 11471837 | 11472826 | 30242.48 | 6.09 | 68.57 | 63.59 | -0.55 | AT5G17810 |
| BrWOX13a | Bra017720 | A03 | 30031149 | 30032217 | 29168.22 | 5.3 | 58.7 | 62.53 | -0.835 | AT4G35550 |
| BrWOX13b | Bra011623 | A01 | 1369091 | 1370429 | 28572.5 | 5.72 | 66.07 | 62.29 | -0.907 | AT4G35550 |
| BrWOX13c | Bra010531 | A08 | 14230412 | 14231397 | 26906.9 | 5.13 | 62.24 | 63.22 | -0.972 | AT4G35550 |
| BrWOX14a | Bra025839 | A06 | 8060028 | 8061118 | 22863.21 | 5.43 | 53.72 | 67.78 | -0.745 | AT1G20700 |
| BrWOX14b | Bra012245 | A07 | 11326741 | 11328832 | 15260.31 | 7.89 | 52.43 | 61.5 | -0.703 | AT1G20700 |
| BoWUSa | Bol012463 | C03 | 29330028 | 29331671 | 33415.68 | 8.23 | 49.76 | 50.1 | -1.002 | AT2G17950 |
| BoWUSb | Bol007486 | Scaffold000260 | 360534 | 362223 | 35383.53 | 6.86 | 57.83 | 47.07 | -1.072 | AT2G17950 |
| BoWOX1a | Bol013842 | C05 | 24742212 | 24744401 | 39825.25 | 8.59 | 51.57 | 48.37 | -1.042 | AT3G18010 |
| BoWOX1b | Bol022905 | C03 | 22864559 | 22866866 | 39972.44 | 7.73 | 55.37 | 50.71 | -1.027 | AT3G18010 |
| BoWOX2a | Bol015487 | C02 | 7962683 | 7963718 | 28548.71 | 9.54 | 55.98 | 56.06 | -0.863 | AT5G59340 |
| BoWOX2b | Bol017313 | C09 | 27161746 | 27163040 | 28632.65 | 9.26 | 47.04 | 57.33 | -0.819 | AT5G59340 |
| BoWOX3a | Bol033227 | C04 | 31741246 | 31742300 | 27260.36 | 9.3 | 72.4 | 64.07 | -0.671 | AT2G28610 |
| BoWOX3b | Bol032947 | C06 | 5304327 | 5305595 | 24977.76 | 9.15 | 63.93 | 64.07 | -0.668 | AT2G28610 |
| BoWOX4a | Bol037032 | C07 | 40023139 | 40024178 | 28917.44 | 9.43 | 61.61 | 60.78 | -0.809 | AT1G46480 |
| BoWOX4b | Bol043226 | C07 | 20302293 | 20303400 | 28526.02 | 9.58 | 66.59 | 57.09 | -0.845 | AT1G46480 |
| BoWOX5 | Bol005895 | C05 | 31560316 | 31561047 | 22471.07 | 8.87 | 58.29 | 60.16 | -0.976 | AT3G11260 |
| BoWOX6a | Bol018879 | C09 | 9432703 | 9434004 | 31891.25 | 5.97 | 51.98 | 63.03 | -1.051 | AT2G01500 |
| BoWOX6b | Bol031538 | C08 | 37570223 | 37571377 | 30176.21 | 5.71 | 71.29 | 52.85 | -1.28 | AT2G01500 |
| BoWOX7a | Bol044014 | C09 | 39003626 | 39004323 | 22099.77 | 8.99 | 47.05 | 62.97 | -0.878 | AT5G05770 |
| BoWOX7b | Bol024490 | C02 | 1887579 | 1888252 | 21745.31 | 8.75 | 48.81 | 64.29 | -0.808 | AT5G05770 |
| BoWOX8 | Bol022811 | C02 | 31477736 | 31479236 | 35542.16 | 6.96 | 49.21 | 71.5 | -0.439 | AT5G45980 |
| BoWOX9a | Bol036992 | C07 | 40559594 | 40561236 | 46672.67 | 7.74 | 61.48 | 67.64 | -0.619 | AT2G33880 |
| BoWOX9b | Bol027305 | C04 | 20597879 | 20599456 | 43232.73 | 8.35 | 64.88 | 67.49 | -0.562 | AT2G33880 |
| BoWOX11a | Bol003826 | Scaffold000339 | 239933 | 241322 | 31984.25 | 5.97 | 74.33 | 54.7 | -0.459 | AT3G03660 |
| BoWOX11b | Bol001196 | Scaffold000461 | 59971 | 61453 | 31714.16 | 6.04 | 60.24 | 66.44 | -0.459 | AT3G03660 |
| BoWOX12a | Bol028614 | C02 | 13108898 | 13110011 | 30970.15 | 6.09 | 69.96 | 57.82 | -0.58 | AT5G17810 |
| BoWOX12b | Bol021367 | C02 | 4394820 | 4395933 | 31119.4 | 5.86 | 70.6 | 60.75 | -0.558 | AT5G17810 |
| BoWOX12c | Bol019774 | C09 | 31913897 | 31914891 | 30547.77 | 5.97 | 70.57 | 62.9 | -0.534 | AT5G17810 |
| BoWOX13a | Bol029067 | C01 | 1798398 | 1799973 | 29259.45 | 5.92 | 63.61 | 65.43 | -0.851 | AT4G35550 |
| BoWOX13b | Bol018704 | C07 | 47114655 | 47115702 | 29059.14 | 5.23 | 65.14 | 62.1 | -0.834 | AT4G35550 |
| BoWOX13c | Bol016371 | Scaffold000156_P2 | 546681 | 547672 | 24987.08 | 5.89 | 64.2 | 67.36 | -0.844 | AT4G35550 |
| BoWOX14a | Bol026982 | C05 | 16142065 | 16143132 | 23619.14 | 5.48 | 55.36 | 66.32 | -0.767 | AT1G20700 |
| BoWOX14b | Bol025459 | Scaffold000087_P1 | 177220 | 178408 | 21686.12 | 5.35 | 62 | 67.03 | -0.588 | AT1G20700 |
| BoWOX14c | Bol025403 | C04 | 38572684 | 38575514 | 33882.61 | 4.68 | 51.62 | 69.52 | -0.505 | AT1G20700 |
| BnAWUSa | BnaA07g02390D | chrA07 | 1993892 | 1995478 | 34305.62 | 6.85 | 51.29 | 47.15 | -1.054 | AT2G17950 |
| BnAWUSb | BnaA06g25450D | chrA06 | 17611881 | 17613553 | 33475.69 | 7.81 | 48.15 | 48.78 | -1.03 | AT2G17950 |
| BnAWUSc | BnaA02g36550D | chrA02_random | 1033782 | 1035437 | 34348.74 | 7.09 | 48.59 | 48.44 | -1.037 | AT2G17950 |
| BnCWUSd | BnaC07g06960D | chrC07 | 11135364 | 11138272 | 34407.93 | 7.36 | 49.61 | 51.66 | -0.985 | AT2G17950 |
| BnAWUSe | BnaA09g09400D | chrA09 | 4699279 | 4701314 | 35861.98 | 6.89 | 52.7 | 46.65 | -1.108 | AT2G17950 |
| BnCWOX1a | BnaCnng51820D | chrCnn_random | 51317648 | 51320085 | 40001.58 | 8.4 | 50.93 | 49.77 | -0.988 | AT3G18010 |
| BnCWOX1b | BnaC03g40380D | chrC03 | 25290549 | 25292598 | 40024.56 | 7.72 | 56.82 | 51.54 | -1.006 | AT3G18010 |
| BnAWOX1c | BnaAnng11050D | chrAnn_random | 12138549 | 12140577 | 40197.58 | 6.97 | 54.46 | 51.1 | -1.04 | AT3G18010 |
| BnAWOX1d | BnaA05g22250D | chrA05 | 17009270 | 17011428 | 40110.87 | 8.59 | 46.39 | 52.25 | -0.936 | AT3G18010 |
| BnCWOX3a | BnaC04g39860D | chrC04 | 40857835 | 40858889 | 27260.36 | 9.3 | 72.4 | 64.07 | -0.671 | AT2G28610 |
| BnAWOX3b | BnaA03g22070D | chrA03 | 10449199 | 10450238 | 26087.02 | 9.33 | 62.76 | 70.31 | -0.604 | AT2G28610 |
| BnAWOX3c | BnaA04g16520D | chrA04 | 13507845 | 13508932 | 27143.24 | 9.32 | 75.63 | 66.28 | -0.669 | AT2G28610 |
| BnCWOX3d | BnaC03g26450D | chrC03 | 15020030 | 15021297 | 25657.64 | 9.26 | 62.38 | 65.41 | -0.618 | AT2G28610 |
| BnAWOX4a | BnaA08g04100D | chrA08 | 3426835 | 3428048 | 29143.63 | 9.31 | 63.31 | 58.63 | -0.84 | AT1G46480 |
| BnCWOX4b | BnaC08g04810D | chrC08 | 5546404 | 5547613 | 28912.43 | 9.43 | 60.4 | 60.39 | -0.817 | AT1G46480 |
| BnCWOX4c | BnaC05g25380D | chrC05 | 20271931 | 20273190 | 28526.02 | 9.58 | 66.59 | 57.09 | -0.845 | AT1G46480 |
| BnAWOX4d | BnaA05g18600D | chrA05 | 13974930 | 13976003 | 28555.1 | 9.63 | 70.99 | 56.33 | -0.847 | AT1G46480 |
| BnCWOX5a | BnaC05g41930D | chrC05 | 39499904 | 39500777 | 22485.09 | 8.87 | 58.29 | 60.16 | -0.976 | AT3G11260 |
| BnAWOX5b | BnaA05g27750D | chrA05 | 19924485 | 19925362 | 22360.95 | 8.87 | 57.15 | 61.68 | -0.958 | AT3G11260 |
| BnAWOX5c | BnaAnng01480D | chrAnn_random | 870586 | 871250 | 21832.34 | 6.66 | 44.65 | 64.29 | -0.801 | AT3G11260 |
| BnAWOX6a | BnaA09g18650D | chrA09 | 11586790 | 11588242 | 30920.26 | 6.41 | 48.16 | 63.6 | -1.003 | AT2G01500 |
| BnAWOX6b | BnaAnng35720D | chrAnn_random | 40487907 | 40488623 | 19173.36 | 8.99 | 48.79 | 71.08 | -0.846 | AT2G01500 |
| BnCWOX6c | BnaC08g39150D | chrC08 | 35002523 | 35004026 | 33988.49 | 5.93 | 70.18 | 57.1 | -1.216 | AT2G01500 |
| BnAWOX6d | BnaA09g45340D | chrA09 | 30993000 | 30994385 | 31961.97 | 6.28 | 56.38 | 48.22 | -1.331 | AT2G01500 |
| BnCWOX7a | BnaC09g49730D | chrC09 | 47953828 | 47954525 | 22099.77 | 8.99 | 47.05 | 62.97 | -0.878 | AT5G05770 |
| BnAWOX7b | BnaA10g24790D | chrA10 | 16128438 | 16129114 | 22114.87 | 9.17 | 51.42 | 63.96 | -0.838 | AT5G05770 |
| BnCWOX7c | BnaC02g02200D | chrC02 | 971477 | 972152 | 21745.31 | 8.75 | 48.81 | 64.29 | -0.808 | AT5G05770 |
| BnCWOX8a | BnaC02g32050D | chrC02 | 34492819 | 34494633 | 35542.16 | 6.96 | 49.21 | 71.5 | -0.439 | AT5G45980 |
| BnAWOX8b | BnaA02g24210D | chrA02 | 17585230 | 17586823 | 35591.17 | 6.96 | 47.23 | 68.16 | -0.485 | AT5G45980 |
| BnCWOX9a | BnaC03g18850D | chrC03 | 9676181 | 9678424 | 44157.46 | 6.57 | 62.05 | 63.6 | -0.626 | AT2G33880 |
| BnAWOX9b | BnaA05g09770D | chrA05 | 5389368 | 5391551 | 43341.87 | 8.35 | 62.33 | 66.51 | -0.557 | AT2G33880 |
| BnCWOX9c | BnaCnng62530D | chrCnn_random | 62338386 | 62340744 | 43232.73 | 8.35 | 64.88 | 67.49 | -0.562 | AT2G33880 |
| BnCWOX11a | BnaC05g48100D | chrC05 | 42761836 | 42763471 | 37085.8 | 5.98 | 58.7 | 83.1 | -0.203 | AT3G03660 |
| BnAWOX11b | BnaA01g34040D | chrA01 | 23027087 | 23028476 | 31778.62 | 6.48 | 68.52 | 64.46 | -0.379 | AT3G03660 |
| BnCWOX11b | BnaC01g40590D | chrC01 | 38800911 | 38802402 | 31778.62 | 6.48 | 68.52 | 64.46 | -0.379 | AT3G03660 |
| BnAWOX11c | BnaA05g32800D | chrA05 | 22415687 | 22417234 | 35661.04 | 6.63 | 67.15 | 75.95 | -0.285 | AT3G03660 |
| BnAWOX11d | BnaAnng41310D | chrAnn_random | 47501056 | 47501989 | 27574.84 | 8.77 | 71.79 | 61.62 | -0.546 | AT3G03660 |
| BnCWOX12a | BnaC02g07490D | chrC02 | 4084005 | 4085345 | 31334.61 | 6.09 | 72.12 | 58.27 | -0.563 | AT5G17810 |
| BnCWOX12b | BnaCnng49570D | chrCnn_random | 48978082 | 48979195 | 31047.23 | 5.97 | 70.55 | 56.79 | -0.585 | AT5G17810 |
| BnAWOX12c | BnaA10g16970D | chrA10 | 12722970 | 12725101 | 30342.52 | 6.34 | 64.47 | 62.99 | -0.61 | AT5G17810 |
| BnCWOX12d | BnaC09g40100D | chrC09 | 42622713 | 42624670 | 30547.77 | 5.97 | 70.57 | 62.9 | -0.534 | AT5G17810 |
| BnCWOX13a | BnaC07g45680D | chrC07 | 43624536 | 43625828 | 29076.23 | 5.23 | 64.29 | 62.34 | -0.826 | AT4G35550 |
| BnAWOX13b | BnaA03g53400D | chrA03 | 28012515 | 28013822 | 29170.28 | 5.3 | 61.74 | 62.97 | -0.829 | AT4G35550 |
| BnCWOX13c | BnaC01g03050D | chrC01 | 1581705 | 1583498 | 29250.4 | 5.67 | 62.99 | 65.43 | -0.851 | AT4G35550 |
| BnAWOX13d | BnaA01g01910D | chrA01 | 970204 | 971701 | 28458.39 | 5.72 | 65.54 | 62.77 | -0.911 | AT4G35550 |
| BnAWOX13e | BnaA08g14960D | chrA08 | 12537105 | 12538086 | 27383.65 | 5.73 | 60.17 | 63.22 | -0.939 | AT4G35550 |
| BnCWOX13f | BnaC03g62360D | chrC03 | 51511015 | 51511712 | 19697.26 | 7.65 | 64.24 | 60.47 | -0.981 | AT4G35550 |
| BnCWOX14a | BnaCnng49040D | chrCnn_random | 48485994 | 48487135 | 23619.14 | 5.48 | 55.36 | 66.32 | -0.767 | AT1G20700 |
| BnAWOX14b | BnaA06g14590D | chrA06 | 7901811 | 7903036 | 22850.21 | 5.43 | 53.86 | 67.78 | -0.732 | AT1G20700 |
| BnCWOX14c | BnaC04g46990D | chrC04 | 46126461 | 46127649 | 21785.26 | 5.51 | 62.99 | 67.03 | -0.609 | AT1G20700 |
| BnCWOX14d | BnaC07g15200D | chrC07 | 21170087 | 21171276 | 21686.12 | 5.35 | 62 | 67.03 | -0.588 | AT1G20700 |
| BnAWOX14e | BnaA07g11310D | chrA07 | 10568807 | 10570838 | 15260.31 | 7.89 | 52.43 | 61.5 | -0.703 | AT1G20700 |
