# Supplementary material for: Genome-wide identification and analysis of the WUSCHEL-related homeobox (WOX) gene family in allotetraploid Brassica napus reveals changes in WOX genes during polyploidization
Source: BMC Genomics. 2019 Apr 25;20:317. doi: 10.1186/s12864-019-5684-3 (PMC6482515; doi:10.1186/s12864-019-5684-3)
Supplement: Supplementary file 2 — Table S2. Estimated Ka/Ks ratios of duplicated WOX gene pairs in B. napus and its diploid progenitors. (DOCX 25 kb) [file 12864_2019_5684_MOESM2_ESM.docx]

**Table S2** Estimated Ka/Ks ratios of duplicated *WOX* gene pairs in *B. napus* and its diploid progenitors.

| Duplicated gene pairs | | | Ks | Ka | Ka/Ks | Duplication type | Types of selection | Time (MYA) |
| --- | --- | --- | --- | --- | --- | --- | --- | --- |
| *BrWUSa* | vs. | *BrWUSc* | 0.3507 | 0.0770 | 0.2196 | Segmental | Purify selection | 11.69 |
| *BrWUSa* | vs. | *BrWUSb* | 0.3033 | 0.0871 | 0.2872 | Segmental | Purify selection | 10.11 |
| *BrWUSb* | vs. | *BrWUSc* | 0.3110 | 0.0846 | 0.2720 | Segmental | Purify selection | 10.37 |
| *BrWOX1a* | vs. | *BrWOX1b* | 0.3336 | 0.0865 | 0.2593 | Segmental | Purify selection | 11.12 |
| *BrWOX3a* | vs. | *BrWOX3b* | 0.2970 | 0.0777 | 0.2616 | Segmental | Purify selection | 9.90 |
| *BrWOX4a* | vs. | *BrWOX4b* | 0.2958 | 0.1014 | 0.3428 | Segmental | Purify selection | 9.86 |
| *BrWOX7a* | vs. | *BrWOX7b* | 0.2845 | 0.0570 | 0.2004 | Segmental | Purify selection | 9.48 |
| *BrWOX9a* | vs. | *BrWOX9b* | 0.3373 | 0.0788 | 0.2336 | Segmental | Purify selection | 11.24 |
| *BrWOX11a* | vs. | *BrWOX11b* | 0.2272 | 0.0729 | 0.3209 | Segmental | Purify selection | 7.57 |
| *BrWOX12a* | vs. | *BrWOX12b* | 0.3738 | 0.0802 | 0.2146 | Segmental | Purify selection | 12.46 |
| *BrWOX13a* | vs. | *BrWOX13b* | 0.3698 | 0.0513 | 0.1387 | Segmental | Purify selection | 12.33 |
| *BrWOX13a* | vs. | *BrWOX13c* | 0.2953 | 0.0858 | 0.2906 | Segmental | Purify selection | 9.84 |
| *BrWOX13b* | vs. | *BrWOX13c* | 0.3234 | 0.0765 | 0.2365 | Segmental | Purify selection | 10.78 |
| *BoWUSa* | vs. | *BoWUSb* | 0.2931 | 0.0853 | 0.2910 | Segmental | Purify selection | 9.77 |
| *BoWOX1a* | vs. | *BoWOX1b* | 0.3185 | 0.0894 | 0.2807 | Segmental | Purify selection | 10.62 |
| *BoWOX2a* | vs. | *BoWOX2b* | 0.3464 | 0.1058 | 0.3054 | Segmental | Purify selection | 11.55 |
| *BoWOX3a* | vs. | *BoWOX3b* | 0.2871 | 0.0634 | 0.2208 | Segmental | Purify selection | 9.57 |
| *BoWOX4a* | vs. | *BoWOX4b* | 0.3070 | 0.1048 | 0.3414 | Segmental | Purify selection | 10.23 |
| *BoWOX7a* | vs. | *BoWOX7b* | 0.3404 | 0.0554 | 0.1627 | Segmental | Purify selection | 11.35 |
| *BoWOX9a* | vs. | *BoWOX9b* | 0.3005 | 0.0901 | 0.2998 | Segmental | Purify selection | 10.02 |
| *BoWOX11a* | vs. | *BoWOX11b* | 0.2210 | 0.0733 | 0.3317 | Segmental | Purify selection | 7.37 |
| *BoWOX12b* | vs. | *BoWOX12c* | 0.3860 | 0.0972 | 0.2518 | Segmental | Purify selection | 12.87 |
| *BoWOX13a* | vs. | *BoWOX13b* | 0.2904 | 0.0579 | 0.1994 | Segmental | Purify selection | 9.68 |
| *BnAWUSa* | vs. | *BnAWUSe* | 0.3468 | 0.0747 | 0.2154 | Segmental | Purify selection | 11.56 |
| *BnAWUSa* | vs. | *BnAWUSb* | 0.3276 | 0.0788 | 0.2405 | Segmental | Purify selection | 10.92 |
| *BnAWUSb* | vs. | *BnAWUSe* | 0.3038 | 0.0829 | 0.2729 | Segmental | Purify selection | 10.13 |
| *BnCWOX1b* | vs. | *BnAWOX1d* | 0.3394 | 0.0922 | 0.2717 | Segmental | Purify selection | 11.31 |
| *BnCWOX3a* | vs. | *BnAWOX3c* | 0.0705 | 0.0203 | 0.2879 | Segmental | Purify selection | 2.35 |
| *BnCWOX3a* | vs. | *BnAWOX3b* | 0.2515 | 0.0646 | 0.2569 | Segmental | Purify selection | 8.38 |
| *BnCWOX3a* | vs. | *BnCWOX3d* | 0.2665 | 0.0701 | 0.2630 | Segmental | Purify selection | 8.88 |
| *BnAWOX3b* | vs. | *BnCWOX3d* | 0.0462 | 0.0324 | 0.7013 | Segmental | Purify selection | 1.54 |
| *BnAWOX3b* | vs. | *BnAWOX3c* | 0.2970 | 0.0777 | 0.2616 | Segmental | Purify selection | 9.90 |
| *BnAWOX3c* | vs. | *BnCWOX3d* | 0.2920 | 0.0751 | 0.2572 | Segmental | Purify selection | 9.73 |
| *BnAWOX4a* | vs. | *BnCWOX4b* | 0.0419 | 0.0207 | 0.4940 | Segmental | Purify selection | 1.40 |
| *BnAWOX4a* | vs. | *BnAWOX4d* | 0.2834 | 0.0925 | 0.3264 | Segmental | Purify selection | 9.45 |
| *BnAWOX4a* | vs. | *BnCWOX4c* | 0.3470 | 0.0966 | 0.2784 | Segmental | Purify selection | 11.57 |
| *BnCWOX4b* | vs. | *BnAWOX4d* | 0.2592 | 0.1076 | 0.4151 | Segmental | Purify selection | 8.64 |
| *BnCWOX4b* | vs. | *BnCWOX4c* | 0.3070 | 0.1088 | 0.3544 | Segmental | Purify selection | 10.23 |
| *BnCWOX4c* | vs. | *BnAWOX4d* | 0.0313 | 0.0102 | 0.3259 | Segmental | Purify selection | 1.04 |
| *BnCWOX5a* | vs. | *BnAWOX5b* | 0.0850 | 0.0136 | 0.1600 | Segmental | Purify selection | 2.83 |
| *BnCWOX7a* | vs. | *BnAWOX7b* | 0.1180 | 0.0273 | 0.2314 | Segmental | Purify selection | 3.93 |
| *BnCWOX7a* | vs. | *BnCWOX7c* | 0.3283 | 0.0554 | 0.1687 | Segmental | Purify selection | 10.94 |
| *BnAWOX7b* | vs. | *BnCWOX7c* | 0.3404 | 0.0554 | 0.1627 | Segmental | Purify selection | 11.35 |
| *BnCWOX8a* | vs. | *BnAWOX8b* | 0.0140 | 0.0081 | 0.5786 | Segmental | Purify selection | 0.47 |
| *BnCWOX9a* | vs. | *BnAWOX9b* | 0.3551 | 0.0970 | 0.2732 | Segmental | Purify selection | 11.84 |
| *BnCWOX11a* | vs. | *BnAWOX11c* | 0.0646 | 0.0368 | 0.5697 | Segmental | Purify selection | 2.15 |
| *BnAWOX11b* | vs. | *BnCWOX11b* | 0.0000 | 0.0000 | - | Segmental | - | - |
| *BnAWOX11b* | vs. | *BnAWOX11c* | 0.2604 | 0.1208 | 0.4639 | Segmental | Purify selection | 8.68 |
| *BnCWOX11b* | vs. | *BnAWOX11c* | 0.2604 | 0.1208 | 0.4639 | Segmental | Purify selection | 8.68 |
| *BnCWOX12a* | vs. | *BnCWOX12d* | 0.3913 | 0.0874 | 0.2234 | Segmental | Purify selection | 13.04 |
| *BnCWOX12a* | vs. | *BnAWOX12c* | 0.3514 | 0.0909 | 0.2587 | Segmental | Purify selection | 11.71 |
| *BnAWOX12c* | vs. | *BnCWOX12d* | 0.0485 | 0.0309 | 0.6371 | Segmental | Purify selection | 1.62 |
| *BnCWOX13a* | vs. | *BnAWOX13b* | 0.1467 | 0.0423 | 0.2883 | Segmental | Purify selection | 4.89 |
| *BnCWOX13a* | vs. | *BnCWOX13c* | 0.3260 | 0.0683 | 0.2095 | Segmental | Purify selection | 10.87 |
| *BnCWOX13a* | vs. | *BnAWOX13d* | 0.2926 | 0.0550 | 0.1880 | Segmental | Purify selection | 9.75 |
| *BnCWOX13a* | vs. | *BnAWOX13e* | 0.3054 | 0.1226 | 0.4014 | Segmental | Purify selection | 10.18 |
| *BnAWOX13b* | vs. | *BnCWOX13c* | 0.3568 | 0.0570 | 0.1598 | Segmental | Purify selection | 11.89 |
| *BnAWOX13b* | vs. | *BnAWOX13d* | 0.3226 | 0.0470 | 0.1457 | Segmental | Purify selection | 10.75 |
| *BnAWOX13b* | vs. | *BnAWOX13e* | 0.2805 | 0.1195 | 0.4260 | Segmental | Purify selection | 9.35 |
| *BnCWOX13c* | vs. | *BnAWOX13d* | 0.1666 | 0.0212 | 0.1273 | Segmental | Purify selection | 5.55 |
| *BnCWOX13c* | vs. | *BnAWOX13e* | 0.3272 | 0.1241 | 0.3793 | Segmental | Purify selection | 10.91 |
